# Essentialome-Wide Multigenerational Imaging Reveals Mechanistic Origins of Cell Growth Laws

**DOI:** 10.1101/2025.06.10.658525

**Published:** 2025-06-22

**Authors:** Daniel S. Eaton, Carlos Sánchez, Luis Gutiérrez-López, Jacob Q. Shenker, Youlian Goulev, Brianna R. Watson, Véronique Henriot, Ethan C. Garner, Quincey A. Justman, Jeffrey R. Moffitt, Johan Paulsson

**Affiliations:** 1 Department of Systems Biology, Harvard Medical School, Boston, MA 02115, USA; 2 Program in Cellular and Molecular Medicine, Boston Children’s Hospital, Boston, MA 02115, USA; 3 Institut Curie, Université PSL, CNRS UMR3348, Orsay, France; 4 Department of Molecular and Cellular Biology, Harvard University, Boston, MA 02138, USA; 5 Department of Microbiology, Harvard Medical School, Boston, MA 02115, USA

## Abstract

Optical Pooled Screening (OPS) enables precise measurement of pooled genetic libraries in single, highly multiplexed microscopy experiments,^1–4^ linking image-based phenotypes to genotypes at genome-scale. However, no method has combined large-scale OPS with multi-generational time-lapse imaging, which is especially important for studying single-cell dynamics and physiology in microbes. Here we develop MARLIN, a technique for multigenerational time-lapse OPS in a microfluidic device and leverage it to map live-cell imaging data to genetic perturbations in a ~130,000-strain mismatch-CRISPRi library targeting all essential genes in *E. coli*. The resulting dataset comprises nearly a hundred million cell cycles and 1.6 billion cells, representing an average of 33 different knockdowns per gene, and provides quantitative measures of morphology and growth for each essential gene with respect to dose. We clustered our multidimensional time-lapse phenotypes to reveal exceptional correlations across co-functional genes, allowing us to systematically discover roles for essential genes of unknown function, as well as implicate RNase E as required for replication initiation. Further, we assayed dose-dependent correlations between growth and cell size for single genes, demonstrating shared regulation of size with ribosome synthesis, while also identifying limitations in existing growth control models. We reconcile these models with our data by positing SpoT as a sensor of translation elongation, which we validate through several lines of genetic and biochemical evidence. Finally, we show that a simple proteome-allocation model – where (p)ppGpp-control only passively limits division protein synthesis – explains all our size-growth scaling behaviors while also quantitatively linking cell size to ribosome abundance. Looking forward, we anticipate that MARLIN will be an essential tool for high-throughput genotype to phenotype mapping in microbes.

Time-lapse microscopy is a powerful tool for cell biology, but is often throughput-limited – finely tracking a few labeled components in a handful of genetic backgrounds, but only managing genome-level screens with great effort^5,6^. Optical Pooled Screening (OPS) promises to change that by leveraging randomly pooled rather than structurally arrayed cell libraries, and then jointly phenotyping and genotyping every cell under the microscope. The randomization reduces batch artifacts when comparing cells, and, by eliminating the need to keep custody of cells in parallel compartments, approaches the physical limit of experimental miniaturization where each individual cell provides a biological replicate. However, current OPS also suffers several limitations. By pooling cells together, it has been difficult to culture and track them over multiple generations of growth, and OPS has therefore focused on single snapshots or short-term dynamics in constant environments. OPS is also challenging^7^ for bacteria and yeast, because of their hard-to-penetrate membranes and low per cell nucleic acid abundance. Microbes would otherwise be exceptionally well suited for multigenerational OPS, thanks to their small physical footprint, short generation times and relative ease of building large, pooled libraries.

Here we present an optical screening platform that combines and extends the key benefits of both arrayed and pooled screens. Specifically, we achieve large-scale OPS for microbes in a microfluidic cell culture format based on so-called “mother machine”^2,8–18^ designs. Cells are loaded as random pools that self-organize into isogenic lineages by single-file growth in trenches, while diffusively fed from continuously replenished media that also serves to evacuate surplus offspring (Fig. 1b). This allows for tracking growth over hundreds of consecutive generations^13,14^ while maintaining exceptionally high cell densities throughout – in local environments that can be finely controlled in time and space^15,17^ – while also minimizing measurement artifacts^15^, allowing for retrieval of cells of interest^16^, and many other features^9,18,2^. Early attempts to expand OPS to this format used Fluorescent In situ Hybridization (FISH) to optically read out RNA barcodes at the end of the experiment.^2,18^ While these were limited to 40 strains per experiment, they laid a foundation that we expand on here, to consider >120,000 strains per experiment, made possible by systematic optimizations:

First, we phenotype hundreds of millions of *Escherichia coli* cell divisions per day, organized into over a million parallel lineages in a dense geometry that enables image acquisition for about ~100,000 lineages per minute (Fig. 1b). The resulting multi-terabyte data volumes are segmented and analyzed using custom software^19^ for parallel computation on HPC systems (Supplementary Methods).

Second, we link the observed phenotypes to genetically encoded barcodes by optically reading out the latter under the microscope. Traditional DNA barcodes of sequence length n can in principle separate between $4^{n}$ variants, but in practice less since the number of nucleotide differences between barcodes (the Hamming distance) must be large compared to the average number of errors inherited from sequencing. Here we instead use an expression-based strategy with n FISH rounds, using labeled probes against unique RNA targets and asking if each target is expressed (“1”) or not expressed (“0”) in a given cell lineage. By using three spectrally distinct probes simultaneously, we in principle separate between $8^{n}$ variants in n rounds of FISH, but in practice less since we ensure that the number of different FISH targets between barcodes (here termed the Herring distance) is much larger than the number of FISH errors. We have heavily optimized this process, placing our barcodes under strong, inducible expression post-phenotyping, extensively screening FISH probes for minimal adsorption into the device polymer, and adapting MERFISH chemistry^1,20,21^ to speed up each probe cycle – typically running 10 rounds of three-color imaging in 14 hours (Methods). To test accuracy, we assayed a pooled library of 120,000 different strains, each expressing a unique 30-bit barcode together with an intact or inactivated copy of a *gfp* gene, with fluorescence providing a ground truth for the *gfp* genotype (Extended Data Fig. 2a,b). This revealed that our FISH barcode read-outs were >99.8% accurate, in a process that is easily automated and costs around $200 per run (Supplementary Methods).

Finally, we link the barcodes to the genetic regions of interest by sequencing both elements on the same read. This requires a challenging combination of long-read sequencing (>1kb) and a low error rate to capture even single point mutations. However, our barcodes help here as well: with typical Herring distances of 3–4 from the nearest barcode, and single-bit Hamming distances of ~10, the whole-barcode Hamming distances are generally >30, providing a clear barcode consensus for every read. Grouping the reads by the barcodes allows us to pinpoint other mutations with great accuracy, e.g. detecting the 2bp substitution distinguishing our *gfp* variants with an accuracy of 99.8% in as few as 20 grouped reads from an R10.3.1 Oxford Nanopore run, with higher accuracy likely possible with recent sequencing chemistries (Fig. 1d and Supplementary Methods).

Taken together, this ‘big FISH’ platform – MARLIN for Multiplexed Assignment of RNA-barcoded LINeages – allows us to precisely track over a hundred thousand strains at the cellular and subcellular levels, each across thousands of individual cells and for dozens of generations of growth in controllable environments, while precisely calling point mutations in a genetic region of interest for each cell. We believe that MARLIN can revolutionize screening in microbes, of anything from perturbation libraries to systematic protein mutants or ensembles of genetic circuits.

## Systematic Measurement of Cell Physiology in an Essentialome-wide Knockdown Library

In our first application of MARLIN, we chose to investigate the contribution of essential genes to cell growth and morphology in *Escherichia coli*. Because loss of essential function leads to inviable cells, we designed our CRISPRi library to include systematically “mismatched” sgRNAs with varying complementarity, and therefore activity, on their targets, manifesting as non-lethal hypomorphic phenotypes.^22–24^ We further measure phenotypes from the onset of knockdown to circumvent the need for long-term cultivation, which also minimizes selection for compensatory mutations in our uninduced pre-cultures.

**Extended Data Fig. 1:**
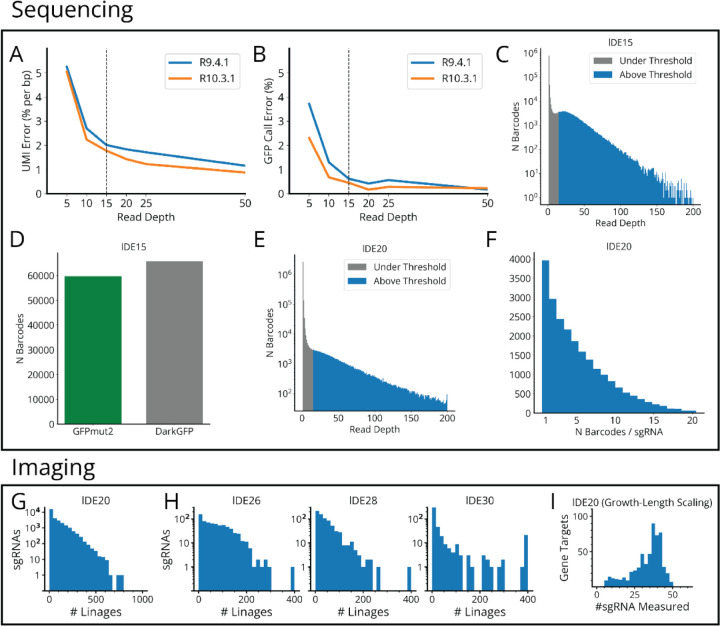
Sequencing Error Rates and Sampling Distributions for MARLIN Experiments. **a,** Error rate (per bp) in the random 15-bp UMI vs read depth, for synthetically down-sampled data. lDE11 (small *gfp* variant library) variants with read depth >200 were sequenced and their consensus UMIs taken as ground truth. Reads assigned to these variants were randomly downsampled to a fixed read depth, re-run through our consensus calling algorithm and compared to the high depth ground truth to estimate the error rate. **b,** Error rate in calling GFPmut2 vs DarkGFP vs read depth, for synthetically down-sampled data generated as in A. **c,** Log scale histogram of the read depth distribution for each lDE15 (large *gfp* variant library) barcode, with a depth cut-off of 15 reads. Grey bars are below the depth threshold and do not contribute to the codebook, while blue bars above the threshold do. **d,** Bar chart showing the number of sequenced barcodes assigned to the GFPmut2 and DarkGFP variants. **e,** Log scale histogram of the read depth distribution for each lDE20 (mismatch-CRISPRi library) barcode, with a depth cut-off of 15 reads. **f,** Histogram of the number of MARLIN barcodes associated with each lDE20 sgRNA sequence. **g,** Histograms of the number of lineages measured for each designed sgRNA in lDE20. **h,** Same histogram for lDE26, lDE28 and lDE30 (HU-mCherry libraries). **i,** Histogram of the number of sgRNAs measured per gene target, for sgRNAs passing the quality threshold (>7 lineages, CV_SEM_< 0.2) for inclusion in our growth-length scaling analysis (Fig. 3–4).

**Fig. 1:**
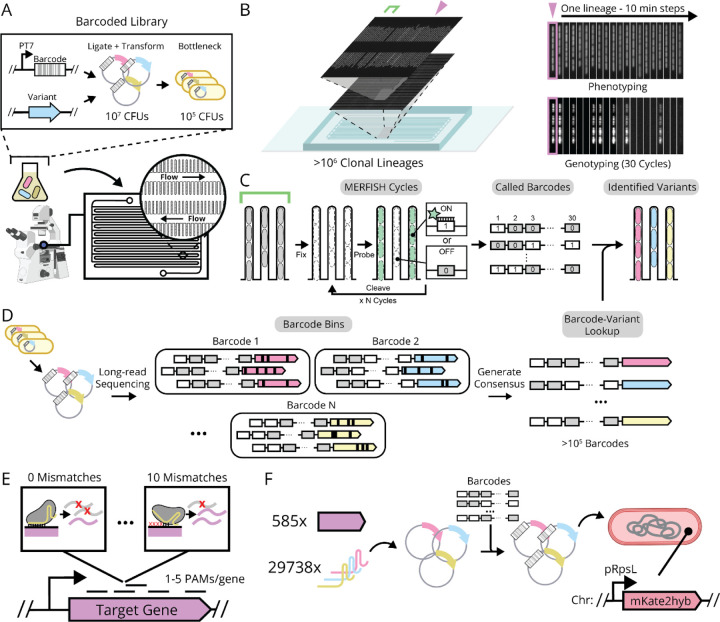
MARLIN - Multiplexed Assignment of RNA-barcoded LINeages. **a,** MARLIN libraries are constructed through combinatorial ligation of MERFISH barcodes to genetic variants and subsequently bottlenecked to ~10^5^ CFUs. During an experiment, libraries are loaded as a pool and cultivated on our high-throughput mother machine. **b,** In a single run, our device enables continuous imaging of >1 million bacterial lineages every 10 minutes, enabling high precision measurement of growth dynamics at scale. **c,** As an endpoint, cells are fixed, and barcodes read using FISH. Dye signal is subsequently eliminated by a disulfide reduction allowing multiple cycles of sequential probing. Decoded barcode sequences are mapped back to their associated library members’ time-lapse data using a sequencing-established lookup table. **d,** The barcode-library variant lookup table is established using nanopore sequencing of our pooled plasmid library. Reads are robustly grouped by FISH barcode in the presence of sequencing-associated error, which is subsequently corrected by consensus averaging for each group. **e,** Our library is designed according to a mismatch-CRISPRi strategy where gene expression is titrated between 0 and 10 mismatches between sgRNA and target. We included mismatch series for up to 5 PAM sites per gene, depending on the number available in the gene. **f,** Our essentialome-wide CRISPRi screen was designed targeting 585 essential genes in *E. coli* with 29738 sgRNAs, derived a previous set of matched sgRNAs^26^, which was barcoded and introduced into MG1655 *E. coli* containing a genomically integrated pRpsL-mKate2Hyb fluorescent reporter.

**Fig. 2:**
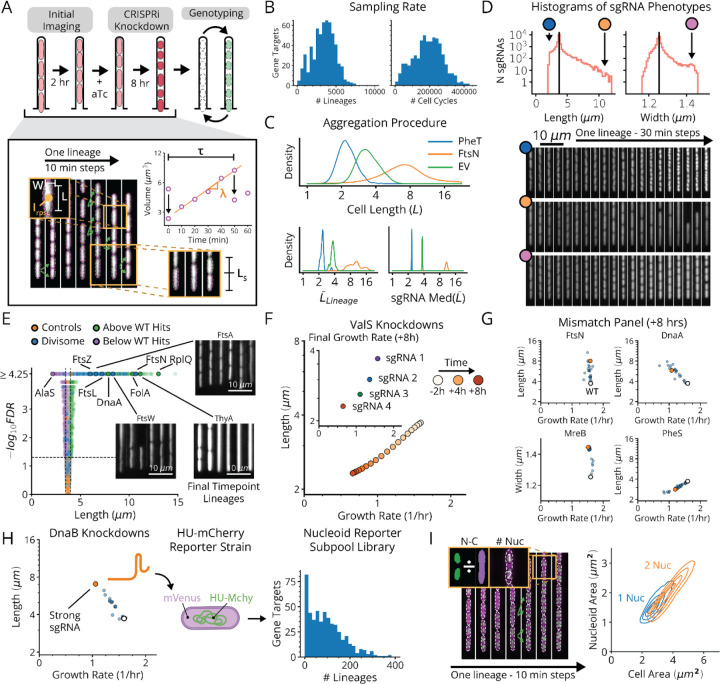
Essentialome-wide Mismatch-CRISPRi with Cell Cycle Measurements. **a,** Top, our CRISPRi library was monitored for over 2 hours of normal growth followed by 8 hours of CRISPRi induction, before being genotyped. Bottom, kymograph of an example lineage from our library, arrows show identified division events. Top-left zoom-in shows that we may quantify length (L), width (W) and pRpsL reporter intensity (I_rpsL_) from single snapshots. Bottom-right zoom-in shows our terminal or “mother” cell growing over time, with its cell volume over time indicated on the graph above. From these data, we may measure both the instantaneous growth rate $\left(\text{λ}\right)$ and the interdivision time $\left(\text{τ}\right)$, as well as the absolute error in septum placement (LS). **b,** Histograms of the number of lineages or cell cycles observed per gene target across all library replicates. **c,** Top, single-cell length distribution for sgRNA variants targeting the indicated genes, between 5–8 hours of CRISPRi induction. Bottom, bootstrap estimator distributions for the length averaged within single lineages (left) and the median of these average lengths, across trenches containing the same sgRNA (right). **d,** Top, log histograms of sgRNA variant average phenotypes, between 5–8 hours of CRISPRi induction. Black line indicates the mean of control sgRNA mean phenotypes. Bottom, representative kymographs of lineages containing sgRNA variants with the indicated mean phenotypes. **e,** Volcano plot of cell lengths measured between 5–8 hours post-induction, for each sgRNA in the library. The horizontal line corresponds to FDR=0.05 and the vertical lines indicate two standard deviations around the distribution of control sgRNAs. Sample lineages are shown of representative and control strains, after 8 hours of CRISPRi induction. **f,** Length vs growth rate plot of a single sgRNA targeting ValS over time. Inset indicates the growth-length scaling of representative ValS sgRNAs after 8 hours of CRISPRi induction. **g,** Length vs growth rate plots for sgRNAs targeting the indicated gene after 8 hours of CRISPRi induction. The white circle indicates the mean length and growth rate of control strains, while the orange dots indicate sgRNAs used in the HU-mCherry reporter library. **h,** Left, length vs growth rate plot for sgRNAs targeting DnaB after 8 hours of CRISPRi induction. A strong sgRNA is selected from the set of DnaB-targeting sgRNAs and pooled with representative sgRNAs targeting all other genes in the library. Right, these sgRNAs were introduced into a strain bearing chromosomally integrated HU-mCherry and pRpsL-mVenus markers. **i,** Left, kymograph of an example lineage from our HU-mCherry library. Top-left zoom-in shows that we may quantify the nucleoid to cell area ratio (NC) and the number of nucleoids (N_n_) in each cell. Right, joint distributions of cell and nucleoid area for single cells with 1 or 2 nucleoids, prior to CRISPRi induction.

Specifically, we designed a CRISPRi library of around 134,000 strains, each with a unique barcode, covering 29,738 different sgRNAs to target 585 essential genes (Fig. 1e,f). Each barcode corresponds to an initial library transformant and so each sgRNA is comprised of on average 4–5 separate clonal lineages, mitigating artifacts from random mutations in lineage founders. We then performed four replicate perturbation experiments, imaging growing lineages for roughly 16 consecutive generations while inducing CRISPRi after 3–4 generations to deplete essential gene products. Technically we could image many more generations, but 16 was chosen for biological reasons, to genotype before all cells in a lineage died from essential gene depletion. In total, ~2 million lineages passed quality controls over the four replicates. This aggregate MARLIN dataset contained at least 200 lineages for 581 of 585 essential gene targets, with ~3,400 lineages observed on average per essential gene (Fig. 2b and Extended Data Fig. 1g).

To capture the global effects of knocking down essential genes, we extracted six phenotypic parameters (Fig. 2a and Methods) from each of the 1.6 billion cell detections included in the MARLIN dataset – cell length (L), width (W), interdivision time $\left(\text{τ}\right)$, instantaneous single-cell growth rate $\left(\text{λ}\right)$, error in division septum placement normalized by cell length (L_s_) and pRpsL fluorescence reporter intensity (I_rpsL_) as a measure of ribosome synthesis regulation. As a control, we imaged growing cells in the absence of CRISPRi for over 40 generations, showing that all measured parameters exhibit stable means and variances over the course of imaging (Extended Data Fig. 3a).

Following CRISPRi induction, our library variants exhibited great phenotypic diversity in every measured parameter across the library (Fig. 2d and Extended Data Fig. 4), producing a gradual spectrum of physiological impacts at different knockdown strengths (Fig. 2f). Quantities were reproducible on repeated measurements of the same sgRNA across regions of the same microfluidic chip, in replicate experiments (Extended Data Fig. 3d) and in select isolates moved into individual non-barcoded strains (Extended Data Fig. 3e). We also verified that well-known biology is captured by MARLIN across genes. For example, members of the Divisome complex, responsible for carrying out cell division, produced significantly increased cell length upon knockdown (Fig. 2e, blue dots). Characteristic phenotypes were also obtained for depletions targeting the Elongasome, the MinCDE system and the ParCE system (Extended Data Fig. 4b–d).

For a subset of sgRNAs, we further used a DNA marker and higher imaging resolution, to better distinguish division and replication defects. Specifically, we selected one or two sgRNAs per gene with a strong effect on cell physiology and introduced these into a strain bearing a HU-mCherry fluorescent fusion protein which localizes to the nucleoid (Fig. 2g,h). This allowed us to quantify both the ratio of the nucleoid to cell area (NC ratio) as well as the number of nucleoids per cell (Fig. 2i). A reduced NC ratio appears to serve as a proxy for the failure to replicate, as exhibited by a DnaA knockdown (Extended Data Fig. 5a). Further, an elevation in the number of nucleoids indicates division failure, as inhibition of division results in the formation of long, poly-nucleated cell filaments, as seen in FtsN knockdowns (Extended Data Fig. 5b). Together our libraries link comprehensive measurements of physiology and replication status to perturbations for essential function, laying the foundation for mechanistic insights into the molecular basis of these traits.

## Clustering Timeseries Phenotypes Reveals Unknown Physiological Functions of Many Genes

Though *E. coli* is arguably the most thoroughly characterized model organism, almost a third of its genes^25^ – including up to 20% of essential genes^26^– encode proteins with unknown function. Homology-based approaches have predicted molecular-level properties for some of these proteins, such as enzymatic activity, but rarely identify the cellular process or phenotypes associated with them.

We therefore asked whether disruptions to the expression of functionally related genes tend to group into similar phenotypes that can be identified by clustering. By quantifying single-cell phenotypes dynamically, separating between initial responses to perturbations and secondary epistatic effects, we reasoned that our approach could result in well-defined clusters for highly specific molecular functions. We could then identify specific functions for poorly characterized genes by examining annotations for those genes they co-cluster with.

**Fig. 3:**
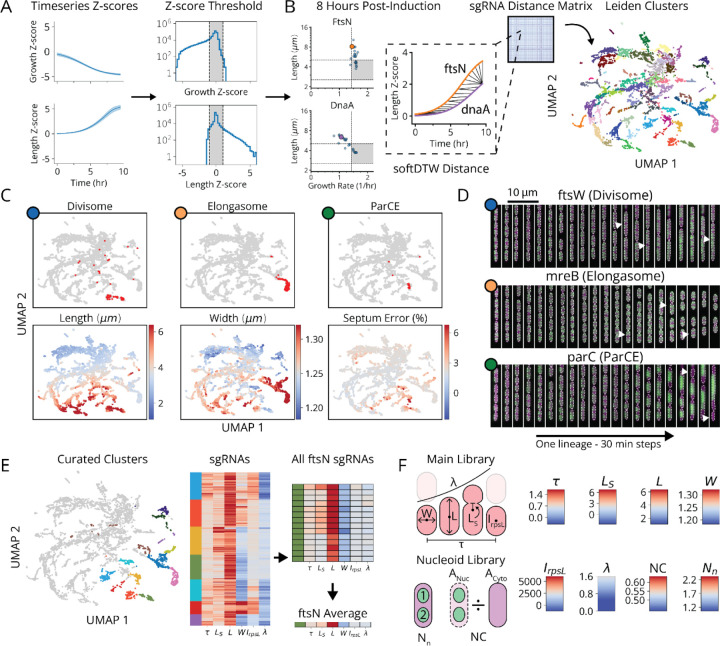
Clustering Reveals Phenotypic Signatures of Essential Processes. **a,** Left, each parameter, measured over time, is converted to a 20-point timeseries using gaussian kernel regression on the data from each trench. These timeseries are converted to Z-scores (Methods) and averaged over trenches belonging to the same sgRNA variant to produce an average timeseries. Right, we subsequently filter out variants with weak phenotypes by excluding those which do not exhibit a Z-score > 1 for any timepoint, along any dimension (grey area). **b,** Conversion of Z-score cutoffs to real values shows where the cutoffs for length and growth rate lie for exemplary genes with size and growth phenotypes. Distances between timeseries data for sgRNA variants are established using a softDTW distance metric, which produces a sgRNA vs sgRNA distance matrix that is then used to generate a 2-dimensional UMAP and clusters based on the Leiden clustering algorithm. **c,** Top, red dots correspond to sgRNAs targeting genes belonging to the indicated class, mapped onto a UMAP representation of each sgRNA variant. Bottom, endpoint values of Length, Width, and Septum Error for each sgRNA. Blue represents values below those of control sgRNAs and red represents values above the controls, with scale given by the right-side bar. **d,** Representative lineage kymographs from our HU-mCherry library, from the indicated classes in Fig. 3c. **e,** Left, subset of clusters associated with a division/replication-like defect, indicated by color. Heatmap indicates average phenotypic quantities after 8 hours of CRISPRi induction for each sgRNA, scale in Fig. 3f. Right, sgRNA variants composing each of our clusters of interest are aggregated into single genes by taking the average of each phenotypic value. Single genes are assigned to the cluster in which the plurality of their targeting sgRNAs can be found, with two clusters assigned in the case of ties. **f,** Top, schematic of quantified variables, see Fig. 2 for details. Bottom, linear scale for variables in Fig. 3e, with red indicating quantities above the EV controls while blue indicates quantities below the controls.

To that end we performed multiparametric clustering to systematically classify CRISPRi knockdowns according to their measured phenotypes.^2,6,27^ To retain information encoded in phenotype dynamics over the course of knockdown, we aggregated measurements for each sgRNA into a multi-dimensional timeseries of Z-scores corresponding to each measured parameter (Fig. 3a and Supplemental Methods). We then generated clusters and a 2-dimensional UMAP based on a distance metric (softDTW)^28^ between those timeseries (Fig. 3b). Following this, each cluster was assessed for statistical stability, measured by comparing cluster membership across jackknife resamples of the same data (Extended Data Fig. 6c). Finally, we collapsed redundant information from different sgRNAs targeting the same gene (Fig. 3e), which usually appear in the same cluster. As an initial validation of this approach, we found that members of multi-protein complexes reproduced phenotypes observed in previous studies and clustered together with their complex partners (Fig. 3c,d).^29–31^

We focused on a set of 36 clusters with strong phenotypes to build a global map of physiology under partial loss of essential function. Strikingly, though these clusters were defined agnostically, based purely on measured phenotypes, we observed an exceptional level of co-clustering for functionally related genes (Fig. 3c and Extended Data Fig. 6d). Integrating gene association, we were able to divide these clusters into five functional supergroups: division, replication, envelope, r-protein and tRNA synthetase. Excluding genes with off-target effects (Supplementary Methods), 100% of Divisome genes were assigned to the division supergroup, 100% of Replisome gene were assigned to the replication supergroup, and 100% of Elongasome genes were assigned to the envelope supergroup. Meanwhile, 93% of sgRNAs targeting r-proteins appeared in the r-protein supergroup and 76% of sgRNAs targeting tRNA synthetases appeared in the tRNA synthetase supergroup. Each supergroup further contained multiple clusters, and these in turn correlated with more specific physiological sub-functions within the supergroup. All five supergroups also contained a minority of other gene depletions, most of which were previously known to be implicated in the supergroup’s associated function, e.g. *minE* which acts as an indirect activator of division.^31,32^ This strongly validates our measurements and analysis, and positions us to annotate genes of unknown function. Leveraging these phenotypic clusters, we associate 13 poorly characterized genes with distinct physiological functions, as well as discover a new molecular factor involved in replication initiation control.

Aside from our multi-gene clusters, five gene depletions in the whole data set did not cluster with others, but were highly reproducible across redundant sgRNAs targeting the same gene, indicating gene-unique phenotypes that could be interesting for follow-up analyses (Extended Data Fig. 6e). Similarly, a few rare exceptions of genes in the same molecular pathways reliably associated with different clusters (Extended Data Fig. 6d). For instance, knockdowns targeting fatty acid or lipid A synthesis enzymes appear in a variety of clusters (Extended Data Fig. 6d) and with a diversity of size scaling behaviors (Extended Data Fig. 7b–d). This suggests that metabolic intermediates in these pathways possess underappreciated activities distinct from those of end products (Supplementary Note S1), e.g. as regulatory players.

The r-protein and tRNA synthetase supergroups were each composed of highly interconnected clusters (Extended Data Fig. 8a,b), with relatively low statistical stability (Extended Data Fig. 6c), reflecting titration of individual genes which populate a phenotypic continuum across these groups (Extended Data Fig. 6a). Nonetheless, many clusters were still enriched for more specifically co-functional genes (Extended Data Fig. 8c), suggesting that finer physiological distinctions could be made in the future with deeper phenotyping. We also observed that the knockdowns targeting the tRNA synthetases for histidine and leucine (*hisS* and *leuS*) had unique phenotypes and occupied single-gene clusters (Extended Data Fig. 6e), demonstrating that single synthetase depletions can exert physiological consequences that are distinct from the general trend. In addition to tRNA synthetases (Extended Data Fig. 8b, orange), we found genes involved in amino acid and fatty acid synthesis, as well as glycolysis, within the tRNA synthetase supergroup. We also found four genes of unknown function within this group – *ysaA*, *ybcF*, *ylbF*, and *yfjT* (Extended Data Fig. 8d). *ybcF* and *ylbF* are consecutive genes on a putative catabolic operon,^33^ suggesting that this uncharacterized metabolism contributes significantly to overall growth.

The remaining three supergroups spanning 13 curated clusters were smaller and more statistically stable, allowing us to further increase the confidence of our functional assignments, by systematically analyzing sgRNAs for evidence of off-target effects using both fitness and cluster phenotypes (Supplementary Methods). The envelope synthesis supergroup includes six distinct and highly stable sub-clusters all involving changes in cell width (Extended Data Fig. 7a, non-underlined). Based on the genes in each group, and consistent with previous data,^30,34^ increased width was associated with outer membrane (OM) or cell wall defects, while decreased width was associated with increased OM stiffness (Supplementary Note S1). Most interestingly, factors implicated in modulating OM composition were found in both wide (*lpxABDH, lptAG, yejM*) and narrow (*waaC, gmhA, rfaE, ftsH*) clusters, implicating the OM as a major determinant of cell width homeostasis. Within the envelope synthesis supergroup, the only gene of unknown function *ykgE* co-clustered with envelope synthesis genes in the high width “Envelope II” cluster shown in Extended Data Fig. 7a, suggesting a role in maintaining envelope integrity.

**Fig. 4:**
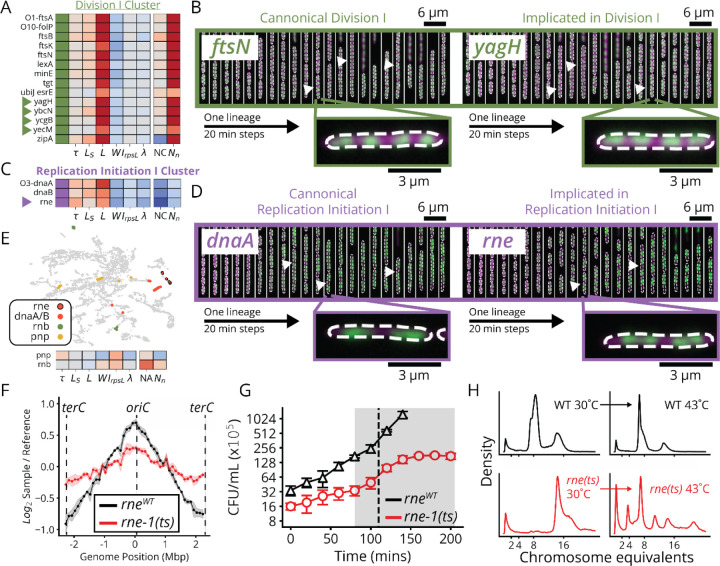
Clusters Implicate Unknown Genes in Division, Replication and Envelope Integrity. **a,** Heatmap of cluster phenotypes for the “Division I” cluster. Arrows indicate genes mentioned in the text. The leftmost column color denotes cluster membership, right columns display the average value of each quantity at the end of the experiment, for each gene. Same scale as in Fig. 3e. Entries beginning with O denote polycistronic operons, where the subsequent gene name indicates the last gene in a consecutive block all belonging to the indicated cluster. See Extended Data Fig. 7 for all curated clusters. **b,** Representative kymographs of genes from the “Division I” cluster, with the cytoplasm in magenta and nucleoid in green, as in the diagram in Fig. 3f. Arrows indicate clear manifestations of the cluster-associated phenotype. Left, kymograph of a *ftsN* knockdown, canonically associated with division. Right, kymograph of a *yagH* knockdown, not previously characterized as division-defective. **c,** Heatmap of cluster phenotypes for the “Replication Initiation I” cluster, same as in Fig. 4a. **d,** Representative kymographs of genes from the “Replication Initiation I” cluster, same as in Fig. 4c. Left, kymograph of a *dnaA* knockdown, canonically associated with replication initiation. Right, kymograph of a *rne* knockdown, not previously characterized as initiation-defective. **e,** Top, colored dots correspond to sgRNAs targeting the indicated genes involved in RNA degradation, mapped onto a UMAP representation of each sgRNA variant. Bottom, heatmap of gene phenotypes for *rnb* and *pnp*. **f,** Marker frequencies based on deep gRNA sequencing for 100kb bins across the *E. coli* genome, with the reference coordinate set to *oriC*. Frequencies normalized to reference measurements from non-replicating cells grown to stationary phase in MBM+0.2% Succinate. Measurements taken after shifting either *rne*^*WT*^ (black) or *rne-1(ts)* (red) cells from 30 °C to 43 °C for 2 hours, with shaded area indicating ±2σSEM based on three biological replicates. **g,** CFU/mL over time for *rne*^*WT*^ (triangles) and *rne-1(ts)* (circles) cells during a shift from 30 °C to 43 °C occurring at 80 minutes (red shading). The vertical dotted line indicates 30 minutes post-shift, the time at which post-treatment samples were taken for replication runout, dividing 1–2 times in this period. **h,** Flow cytometric measurement of PicoGreen-stained DNA before (left) and after (right) replication runout for cells grown first at 30 °C and shifted to 43 °C for 30 minutes, in strains bearing either *rne*^*WT*^ (black) or *rne-1(ts)* (red) alleles. *rne-1(ts)* cells drop from 16 to 4–8 oriC copies after 30 minutes at 43 °C, indicating that initiation immediately halts on temperature shift and the original 16 oriC have simply partitioned into either 2 or 4 descendent cells.

The cell division supergroup included three distinct and highly stable sub-clusters, characterized by an increase in cell length, without any change in growth (Fig. 4a and Extended Data Fig. 7a, Underlined and Unbolded), consistent with previous findings for partially division-defective cells.^35^ Integrating our nucleoid imaging data, we confirmed that the “Division I” cluster shown in Fig. 4a exhibited clear division-specific defects, with filamentous, polynucleate phenotypes (Fig. 4a,b). The genes of unknown function among this group (*yagH*, *ybcN*, *ycgB* and *yecM*) similarly exhibit this phenotype (Fig. 4a), implying impaired division but no replication defect. The other clusters, while division defective, exhibited more complex nucleoid and morphological phenotypes (Extended Data Fig. 7a). As discussed in Supplementary Note S1, these clusters likely impair division indirectly through either reduced lipid synthesis (“Division II” cluster) or dysregulated division localization (“Septum Error” cluster), and include several genes of unknown function (*yeiH*, *yfaU*, and *yhcN*).

The DNA replication supergroup contained four distinct sub-clusters (Fig. 4c and Extended Data Fig. 7a, Underlined and Bold). Like the division-defective clusters, this group exhibited an increase in cell length, but were instead distinguished by a decreased growth rate, consistent with recent reports.^36^ Remarkably, our replication-associated subclusters were primarily distinguished from each other by cell width, and each associated with a specific function in replication (Fig. 4c and Extended Data Fig. 7a, Underlined and Bold). Based on the genes in each of the four clusters, we successfully associated these groups with either replication initiation, dTTP synthesis or the Replisome itself. Broadly, members of these clusters are either directly involved in replication, or indirectly involved through impacts on transcription or transcription termination that have previously been shown to affect replication (Supplementary Note S1). We found that genes with a direct role in replication exhibit a decrease in nucleoid extent, consistent with their function, while those with an indirect role through transcription exhibit an increase in extent, which has indeed been observed when treating cells with transcription-inhibiting drugs.^37,38^ Among these indirect hits was the only gene of unknown function in this supergroup *yejF*, though its role remains ambiguous since its sgRNAs are split evenly between replication and envelope-related clusters (Extended Data Fig. 7a).

Interestingly, our replication initiation-related clusters suggested a previously unappreciated player in this textbook molecular process. Replication initiation has been extensively characterized both *in vivo* and *in vitro*, with three core factors, DnaA, DnaB and DnaC, participating in opening the replication bubble at the origin of replication (*oriC*).^39^ Strikingly, we detected one replication initiation cluster consisting of only these three proteins as well as one other well-studied gene: the single-strand endoribonuclease RNase E, encoded by *rne*, which is not known to play a role in this process (Fig. 4c,d). Interestingly, RNase E does not co-cluster with either of the 3’ to 5’ ssRNA exonucleases RNase II (*rnb*) and PNPase (*pnp*) (Fig. 4e), indicating that its phenotype does not result from generic loss of mRNA degrading activity. To test whether RNase E activity is required for replication initiation, we used the fact that halting initiation reduces ploidy near *oriC*. Specifically, using a temperature-sensitive RNase E allele, we confirmed that the relative *oriC* copy number dropped within a few minutes of RNase E inactivation, measured by deep sequencing (Fig. 4f and Extended Data Fig. 9b), while a replication runout assay (Fig. 4h, *bottom* and Extended Data Fig. 9c–e) indicated immediate loss of initiation activity. RNase E is thus required for the initiation of DNA replication *in vivo*, demonstrating that our approach can reveal new essential functions for even the most well-studied genes involved in the most central processes in *E. coli*.

## Growth Control in *E. coli* Depends on Translation Elongation

When cells are perturbed, whether externally by changing conditions or internally by genetic modifications, their phenotypic responses can follow distinct quantitative patterns. For example, the classic ‘nutrient growth law’ of Schaechter, Maaløe, and Kjeldgaard (SMK) shows that cell size approximately scales exponentially with growth rate for a wide range of external changes in nutrient levels.^40^ Ribosomes in turn obey a famous linear growth law where faster growth produces proportionally more ribosome expression^41^. Both laws are enforced by the regulatory action of the small molecule alarmone (p)ppGpp, which represses both size and ribosome synthesis.^42,43^ Our methods above allow us to more comprehensively explore such phenotypic patterns by comparing growth and size for tens of thousands of internal perturbations arising from graded loss of gene expression. Strikingly, this reveals three distinct variants of each growth law. Here we describe these phenotypically, explain how they arise from two distinct translation-mediated mechanisms for (p)ppGpp control, and propose a unified mechanistic and quantitative explanation for all variants.

**Fig. 5:**
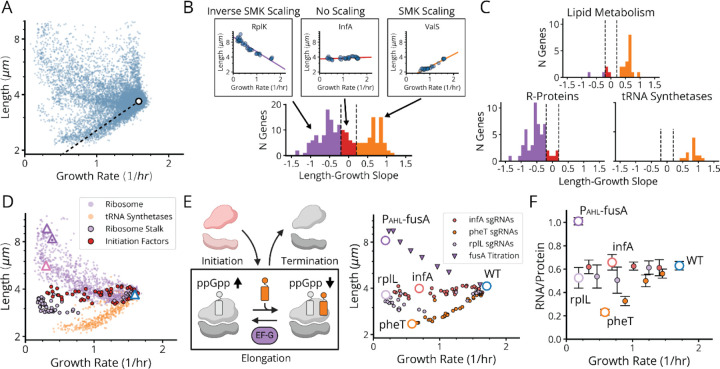
Three Size-Growth Scalings Emerge from Translation Knockdowns. **a,** Length vs growth rate for each sgRNA variant averaged between 5–8 hours after CRISPRi induction. Dashed line a guide for the log-linear decrease in length with growth rate. **b,** Top, select log2 length vs growth rate linear fits to groups of sgRNAs targeting single genes. Bottom, histogram of the log2 length vs growth rate slope for genes exhibiting at least one sgRNA with a growth rate less than 0.9 doublings per hour. Colors correspond to slopes less than −0.2 (purple), greater than 0.2 (orange) and between −0.2 and 0.2 (red). **c,** Histograms of log2 length vs growth rate slope for indicated gene groups. **d,** Length vs growth rate averaged between 5 and 8 hours after CRISPRi induction for variants corresponding to ribosomal proteins/rRNAs (purple circles), tRNA synthetases (orange circles), ribosome stalk proteins L7/L12 and L10 (black outlined purple circles) and initiation factors (black outlined red circles). Initiation factor InfC was omitted due to likely disruption of downstream gene expression. Note that ribosome stalk knockdowns exhibit exceptionally weak size scaling compared to other r-proteins (see Fig. 6c). Triangles correspond to points for which individual isolates were cloned in E, including 50S subunit proteins RplQ and RplA (purple), the 30S subunit protein RpsO (pink) and an EV control sgRNA (blue). **e,** Left, simplified kinetic model of translation, consisting of initiation, elongation and termination. Elongation kinetics are coarse-grained into an uncharged state (empty A-site) and a charged state (charged tRNA in the A-site). Colors indicate gene functions targeted in the corresponding knockdown strains. Right, small circles indicate average length vs average exponential growth rate for the indicated genes after 5–8 hours of CRISPRi induction (pheT, infA, rplL) in lDE20. Inverted triangles indicate the same values for a strain with FusA under AHL induction (P_AHL_-fusA), 5–8 hours after stepping down from an AHL concentration of 1 μM to 0.2 μM, 0.16 μM, 0.13 μM, 0.1 μM, 0.07 μM, 0.05 μM, or 0.03 μM. Large circles represent the same values for indicated CRISPRi strains (DE428, DE830, DE831, and DE832) induced by aTc or the AHL induction strain (DE828) stepped down to 0 μM AHL. **f,** Average RNA/Protein ratio (n=5–6) after 6 hours of CRISPRi induction or removal of AHL vs average exponential growth rate from Fig. 5e. Large circles indicate the same strains and knockdown conditions as in the large circles in Fig. 5f and Fig. 6b. Error bars denote 2σ_SEM_ range.

**Fig. 6:**
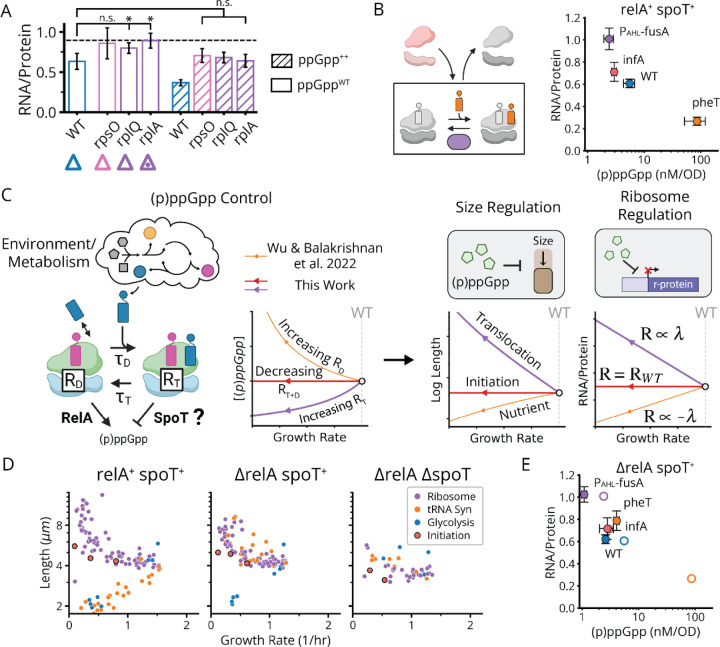
(p)ppGpp Degradation by Translocating Ribosomes Reconciles Diverse Translation Phenotypes. **a,** RNA/Protein ratio after 6 hours CRISPRi depletion of indicated genes. WT denotes the EV sgRNA control. Unhatched bars indicate cells uninduced for the hyperactive (p)ppGpp synthase RelA* and hatched lines indicate +25uM IPTG to induce RelA* expression, resulting in ectopic synthesis of (p)ppGpp. Error bars denote 2σ_SEM_ range. Bars indicated with “*” exhibit a significant increase in RNA/Protein relative to WT (p<0.05). Dotted line indicates the maximum RNA/Protein ratio (~0.89) reported in a previous study limiting protein synthesis by chloramphenicol.^69^ **b,** Left, same schematic as in Fig. 5e. Right, RNA/Protein and (p)ppGpp measurements of the indicated sgRNA knockdowns, P_AHL_-fusA and an EV sgRNA control (WT), in a *relA*^*+*^ *spoT*^*+*^ background. All measurements made after 6 hours of CRISPRi induction or removal of AHL. Error bars denote 2σ_SEM_ range. **c,** Left, illustration of a two-step model of elongation. Production of (p)ppGpp in proportion to uncharged or “dwelling” ribosomes $\left({\text{R}}_{\text{D}}\right)$ is mediated by RelA, as previously described.^47^ Degradation of (p)ppGpp promoted by charged or “translocating” ribosomes $\left({\text{R}}_{\text{T}}\right)$, in a SpoT-dependent manner, is proposed to reconcile our observations. Constitutive (p)ppGpp production by SpoT is assumed insignificant in WT cells, but considered as the only productive term in *ΔrelA spoT*^*+*^. Right, model-predicted changes in (p)ppGpp, cell length, or RNA/Protein vs growth rate under increased dwell time (orange), increased translocation time (purple) or reduced translation initiation (red). Note the prediction for cell length is qualitative, since the (p)ppGpp to length function is not modeled. The model also accounts for RNA/Protein and size invariance under *rplL* knockdown, which interferes with initiation, translocation and charged tRNA capture (Supplementary Theory).^70^ **d,** Average length vs average exponential growth rate for translation-related variants after 5–8 hours of CRISPRi induction in *relA*^*+*^ *spoT*^*+*^*, ΔrelA spoT*^*+*^*, ΔrelA ΔspoT* backgrounds. Ribosomal proteins are purple, tRNA synthetases are orange, glycolysis enzymes are blue, and initiation factors are red. **e,** RNA/Protein and (p)ppGpp measurements of the same knockdowns as in Fig. 6a, in a *ΔrelA spoT*^*+*^ background. Empty circles indicate values in *relA*^*+*^ *spoT*^*+*^.

First, many gene knockdowns (65 of 185) with a growth defect (<0.9 doublings/hour) conform to classical SMK scaling, including tRNA synthetases, central metabolic enzymes and fatty acid biosynthesis genes (Fig. 5a–d, Extended Data Fig. 10c). This supports our understanding of how translation affects (p)ppGpp: under conditions of low tRNA charge, there are more elongating ribosomes with uncharged tRNAs in their A-site,^44,45^ where the enzyme RelA can bind to stimulate its synthesis of (p)ppGpp. In a separately cloned knockdown of the tRNA synthetase PheT, we used LC-MS/MS to directly confirm that (p)ppGpp indeed was strongly elevated (Fig. 6b) and measured the RNA/Protein ratio to confirm that ribosomes were correspondingly downregulated (Fig. 5f).

Second, 90 genes in our dataset – including 41 of the 45 strongly growth-inhibiting ribosomal protein (r-protein) knockdowns (Fig. 5c,d, purple bars) – instead *inverted* the SMK growth law and produced negative scaling between cell size and growth rate. This seems to support the same tRNA charge explanation as above: knockdowns of individual r-proteins often compromise ribosome elongation^46^ and decrease tRNA consumption, which increases tRNA charge while reducing RelA activity and (p)ppGpp levels. We also observed the accompanying inversion in ribosome scaling with increased RNA/Protein at lower growth rates (Fig. 6a), and found that ectopic expression of a hyperactive (p)ppGpp synthase (RelA*) largely restores size and ribosome content to wildtype levels (Fig. 6a and Extended Data Fig. 5g). However, we then identified a third scaling class where knockdowns targeting the few remaining r-proteins, located in the ribosome stalk, as well as translation initiators *infA* and *infB*, form a group that exhibit flat scaling with *no discernable correlation* between size and growth (Fig. 5d, Extended Data Fig. 10b). Both (p)ppGpp and ribosome levels in an *infA* knockdown are indeed similar to wildtype (Fig. 5f).

This is an important discrepancy: by our expectations, knocking down initiation factors should have mimicked r-protein knockdowns and similarly reduced the consumption of charged tRNA. The fact that there is a distinct difference suggests that the inverse SMK scaling for r-protein knockdowns is *not* in fact due to altered tRNA charging in general, but to a more specific step. We therefore analyzed knockdowns of the EF-G protein – specifically targeting EF-G-mediated ribosome translocation. The results closely mirrored the effects of knocking down r-proteins: decreased growth, decreased (p)ppGpp, increased size and an increased RNA/Protein ratio (Fig. 5e,f, 6b)^47–49^. This further shows that initiation and translocation differentially affect (p)ppGpp levels, suggesting additional control mechanisms beyond RelA. Next we explore those mechanisms.

## Ribosomes and Cell Size Respond to Translation Elongation Defects via SpoT and (p)ppGpp

A recent mathematical analysis^47^ of how (p)ppGpp levels quantitatively scale with elongation rate suggested that translating ribosomes play a direct regulatory role, not just in (p)ppGpp production via RelA, but also by making the (p)ppGpp degradation rate proportional to the number of ribosomes carrying a charged A-site tRNA i.e. that are primed to translocate. Such a mechanism would diminish (p)ppGpp in response to reductions in translocation rate, but will likely not respond to initiation defects – which should only weakly impact the translocation rate in rich conditions i.e. where this rate is already maximized – as we observed both for the libraries and in the targeted experiments focused specifically on pure initiation and translocation knockdowns. This prompted us to look for such a translocation-specific regulator.

The only known (p)ppGpp regulator in *E. coli*, apart from RelA, is the bifunctional enzyme SpoT which both constitutively synthesizes and degrades (p)ppGpp^50^. We therefore hypothesized that SpoT provides the translocation-specific (p)ppGpp hydrolysis responsible for the inverse SMK scaling under inhibited translocation. To test this, we sought to compare CRISPRi libraries with and without *spoT*. While the single *ΔspoT* knockout does not grow, due to the stabilization and accumulation of lethal amounts of (p)ppGpp synthesized by RelA^51^, the *spoT* deletion can be made in a *ΔrelA* strain. We therefore compared growth and size patterns across three CRISPRi libraries – the WT above as well as both the double knock-out *ΔrelA ΔspoT* and the single knockout *ΔrelA*.

In the *ΔrelA ΔspoT* background, which have no (p)ppGpp^15^, both the SMK scaling for tRNA synthetase knockdowns and the inverse SMK scaling for r-protein knockdowns collapse to flat lines, like the initiation factor knockdowns. This confirms that the scaling laws depend on (p)ppGpp. We then considered the single *ΔrelA* knockout where cells have the SpoT activity added back, which should restore both its constitutive (p)ppGpp production activity^51^ and its putative translocation-dependent hydrolysis. Strikingly, we find that adding back SpoT completely restores the inverse SMK scaling under r-protein knockdown observed in the wildtype (Fig. 6d). We also confirm that this response is specific to translocation defects, by demonstrating similar effects for an EF-G knockdown in *ΔrelA* strains which produce a wildtype-like increase in ribosome levels and reduction in (p)ppGpp (Fig. 6e). Similar results hold for initiation defects, where again as in wildtype cells, size and ribosome levels are unaffected in a *ΔrelA* background (Fig. 6d,e).

In contrast to wildtype, however, in the *ΔrelA* strain the tRNA synthetase knockdowns *do not* follow SMK scaling, but rather *inverse* SMK scaling (Fig. 6d). Similarly, an inverted (p)ppGpp response to single amino acid auxotrophy has been observed previously^50^, which has not been accounted for thus far. This, we argue, further supports a SpoT-dependent translocation response, as these knockdowns are predicted to impede translocation due to ribosome queuing upstream of codons with undercharged cognate tRNAs (see Supplementary Theory). Consistent with this explanation, knockdowns which inhibit the synthesis of multiple amino-acids, and should thus not produce excessive queueing, do not exhibit this inverse scaling in *ΔrelA* (Fig. 6d). Therefore, RelA appears to mask what would otherwise be an inverted, SpoT-mediated response to single amino acid depletion in WT – showing that our proposed mechanism also clarifies previously unintuitive results. These data show that *spoT* is necessary for the reduction of (p)ppGpp under reduced translocation, while *relA* is required for standard SMK scaling under reduced tRNA synthetase activity.

Finally, we show that a combined RelA/SpoT model explains the remarkable linearity of ribosome-growth scaling, under varying nutrient quality, elongation inhibitors and translation initiation defects (Fig. 5f).^41,52,53^ Previously, linear ribosome-growth scaling was theoretically suggested to arise as a consequence of maximizing overall translation flux^53^ through control of ribosomes. We show that even without maximizing translation flux, the same behavior also emerges from the proposed (p)ppGpp control model – assuming simple, first-order kinetics in (p)ppGpp production and degradation. Such a model produces positive-linear ribosome scaling under reduced tRNA charge (Fig. 6c, orange line) – previously derived elsewhere^47^, negative-linear scaling under reduced elongation (Fig. 6c, purple line) and invariance under reduced translation initiation (Fig. 6c, red line) (see Theory Box).

Briefly, the previous derivation^47^ of positive-linear ribosome scaling under reduced tRNA charge follows from the inverse scaling of the (p)ppGpp concentration g with the elongation rate ε under these conditions. In particular, $g\propto (\varepsilon_{Max}/\varepsilon )-1$ where $\varepsilon_{Max}$ is the maximum elongation rate, which was subsequently empirically validated. Continuing, the authors assume (p)ppGpp control of ribosome levels R is given by a simple inverse function R∝1/g, while the growth rate λ itself depends on the product of ribosome levels with the elongation rate λ=εR. To capture the accumulation of inactive “hibernating” ribosomes H at high *g*, the authors inserted a corrective term – resulting in λ=ε(R−H). The measured scaling of hibernating ribosomes with (p)ppGpp^47^ subsequently motivated the form H∝g. Solving this system of equations for λ(R) results in a complex form. However, under the simplifying, empirically verified assumption that the minimum elongation rate $\varepsilon_{0}$ is half $\varepsilon_{Max}$, the authors obtained the observed positive-linear scaling $\lambda \propto -R_{0}+R$.

Following a similar approach, we show that linear model behavior can also be obtained for the reduced translocation and initiation regimes. Beginning with reduced translocation, we note that for this perturbation, the (p)ppGpp concentration g instead scales positive-linearly with ε. Here, we model (p)ppGpp control of ribosome levels R by a simple monovalent repressor function R∝k/(k+g), while λ=ε(R−H) still holds. However, we instead treat hibernating ribosomes as a fixed value $H_{0}$, motivated by the observed invariance in this value except at low growth rates.^54^ We subsequently note that $H_{0}$ and the minimum ribosome expression level $R_{0}$ take similar values – allowing us to use the simplifying assumption $H_{0}=R_{0}$. Along with the proportionality between elongation rates and (p)ppGpp levels, this allows us to obtain $g\propto \lambda /\left(R-R_{0}\right)$. Finally, we may rearrange the ribosome repressor function to get $g\propto \left(R_{max}-R\right)/\left(R-R_{0}\right)$. Since both ribosome levels and growth rate are scaled to (p)ppGpp levels by the same inverse dependence on $R-R_{0}$, it follows that growth rate and ribosome levels obey a negative-linear relationship $\lambda \propto R_{max}-R$ as observed.^52^ The remaining invariant scaling under initiation factor depletion (Fig. 6c, red line) proceeds simply from the expectation that neither dwelling nor translocation times will change. That is, under the rich growth conditions examined here, ternary complex diffusion should be limiting for the dwelling step^55^ and increases in tRNA charge from lower translation flux should not impact dwelling. Similarly, the translocation rate is likely near-maximum under these conditions^54^ and so increases from i.e. reduced ribosome queueing are unlikely. As such, ribosome levels should remain fixed with respect to growth under translation initiation defects, as we observe (Fig. 5f). Thus, our model for the combined RelA-SpoT system largely accounts for the remarkably robust linearity between ribosomes and growth across diverse translation perturbations.

## The Nutrient Growth Law is Negative Hyperbolic and Emerges from Optimal Protein Synthesis

Above we showed how translation-mediated (p)ppGpp control creates the linear scaling of ribosomes with growth rate, for all three classes of perturbations observed. Next we seek to explain the nutrient size growth law. Because cell volume responds to growth in a (p)ppGpp-dependent manner, and (p)ppGpp directly controls transcription of many genes, it is tempting to hypothesize that some key division protein is under direct transcriptional control of (p)ppGpp. However, to our knowledge no such candidates have been identified, and most known division proteins are instead demonstratively *not* regulated by (p)ppGpp.^56^ Here we propose that not only the classical SMK growth law, where volume seems to depend exponentially on growth, but all three variants observed above, precisely reflect this *absence* of direct (p)ppGpp control of division proteins.

Specifically, our model postulates that cells on average divide after accumulating enough of some unspecified set of ‘division proteins’ that are not directly controlled by (p)ppGpp, whether structurally involved in division or in some indirect regulatory process. This assumption is supported by the observation that most division-related promoters are indeed not regulated by (p)ppGpp^56,57^ and is consistent both with *E. coli*’s behavior as a division ‘adder’^58,10^ where cells correct size deviations regressively by adding constant mass regardless of deviations in birth size, and the fact that changes in the expression of division proteins correlate with changes in cell size.^59^ In the simplest model, the average volume V of cells is then inversely proportional to the fraction or ‘sector’ $\phi_{NC}$ of the proteome not controlled by (p)ppGpp, which in turn is complementary to the fraction $\phi_{C}=1-\phi_{NC}$ that *is* controlled by (p)ppGpp, i.e., $V\propto 1/\left(1-\phi_{C}\right)$.

**Fig. 7:**
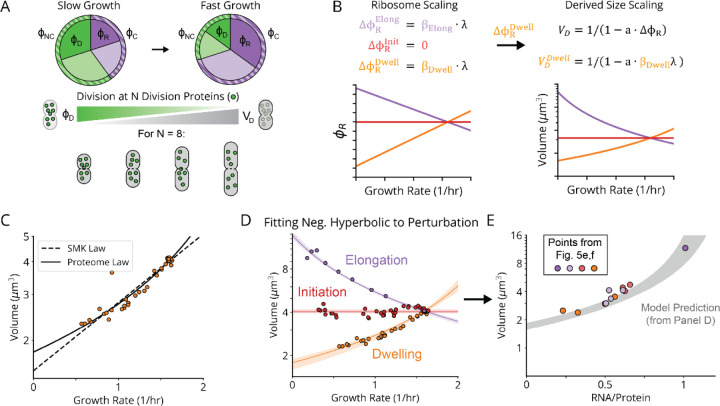
Size Scaling Law Emerges from Proteome Distortion by Ribosome Expression. **a,** Illustration of our size scaling model. The proteome is divided into two coarse-grained sectors, $\phi_{\text{C}}$ containing proteins repressed by (p)ppGpp and $\phi_{\text{NC}}$ containing proteins that are not directly regulated by (p)ppGpp. $\phi_{\text{C}}$ obeys the same regulation as ribosomal proteins and therefore the proteome fraction made up of ribosomes $\phi_{\text{R}}$ is a subset of this fraction. Division is assumed to occur when a subset of proteins in $\phi_{\text{NC}}$, with a fractional abundance of $\phi_{D}$, reach a threshold abundance D*. **b,** Model-derived scaling of the ribosome proteome fraction $\phi_{\text{R}}$ (left) and cell volume (right) with growth rate, under decreasing translocation rate (purple), initiation rate (red) and ternary complex capture rate (orange). **c,** Similar measurements as in Fig. 5e, for *pheT* knockdowns, with cell volume estimated from length and width assuming the cell has a spherocylindrical shape. Lines correspond to fits to the negative hyperbolic and exponential (i.e. SMK) size models. **d,** Same measurements as in Fig. 7d, for *pheT* (orange), *infA* (red) and *fusA*/EF-G (purple) knockdowns. Lines correspond to a three parameter, joint fit of the negative hyperbolic to *pheT* and *fusA* knockdowns, with the trendline for *infA* given by the point of intersection. 95% confidence intervals were determined by residual bootstrapping. **e,** Datapoints correspond to the empirical RNA/Protein vs volume relationship, derived from the data in Figs. 5e–f. Shaded area corresponds to the 95% confidence interval of the model-predicted relationship, given the fit in Fig. 7e.

We further postulate that the sector $\phi_{C}$ controlled by (p)ppGpp varies linearly with the ribosome sector, i.e., $\phi_{C}=a_{1}\phi_{R}+b_{1}$. This is roughly true simply because ribosomes constitute a large part of this sector. However, linearity also follows exactly if (p)ppGpp controls all (p)ppGpp-regulated promoters by the same factor, which seems likely since it regulates expression by binding RNA polymerase far from the DNA binding domains, minimizing promoter-specific effects (see Theory Box). We experimentally validate that, across many growth conditions, the (p)ppGpp-repressed fraction of expression varies linearly with the ribosomal protein fraction, at both the protein and mRNA levels (Extended Data Fig. 11).^57,60,61^ This predicts a volume-ribosome relationship of the form $V\propto {\left(1-c\phi_{R}\right)}^{-1}$ where c is a positive constant, as also directly supported by our measurements across different knockdowns (Fig. 7e, white dots). Finally, we connect the ribosome sector $\phi_{R}$ to growth rate λ by using the linear ribosome growth law above, $\phi_{R}=a_{2}\lambda +b_{2}$ with different slopes under defects for tRNA charging, translocation and initiation, a form we and others have experimentally verified (Fig. 5f, data pending for *fusA*).^53^

These simple and experimentally supported assumptions predict a negative *hyperbolic* relationship between volume V and growth rate λ, i.e., on the form $V∼{\left(1-k\lambda \right)}^{-1}$ rather than the classic exponential $V∼e^{k\lambda }$. For the SMK perturbation class, the hyperbolic curve strikingly fits data at least as well as the classical exponential curve, even though the exponential was chosen based on fitting (Fig. 7c) and the hyperbolic is predicted from underlying mechanisms. In fact, the hyperbolic function quantitatively captures all three perturbation classes (Fig. 7d), with class-specific *size scaling* constants strictly proportional to their underlying *ribosome scaling* constants, consistent with our mechanistic expectations (Extended Data Fig. 12b). Together, our evidence shows that, despite describing different phenotypes and taking different quantitative forms, both the nutrient and ribosome growth laws arise from (p)ppGpp-mediated regulation of ribosome synthesis.

For maximal generality we refrain from proposing a specific model for the inverse proportionality between division volume and division protein levels. However, we do note that the assembly of the division-mediating Z-ring, which requires sufficient division protein to span the perimeter of the mid-cell cross-section, could mediate such a proportionality. Indeed, we observe that division volume exhibits a positive, roughly linear dependence on the mid-cell perimeter for the knockdowns that alter cell width (Extended Data Fig. 12a), and that applying a perimeter-based correction to our size-growth scaling model also results in a high-quality fit (Extended Data Fig. 12c).

It has been a long-standing mystery why so many bacteria follow the nutrient growth law of approximately exponential size-growth scaling.^10,40,62^ Our results show that the exponential is similar to the hyperbolic, which arises naturally from simple criteria that could be satisfied in many organisms. Specifically, the linear relation between ribosome levels and growth rate, the complementary linear decrease in protein sectors not co-regulated with ribosomes, and the lack of strong co-regulation between division and ribosomes, may all hold broadly. Many other cell processes could similarly respond to changes in conditions in a seemingly controlled and coordinated manner, simply by *not* being directly regulated by (p)ppGpp or its equivalents. In fact, properties that stay *constant* across conditions are the ones more likely to be under (p)ppGpp control. For example, the initiation volume per origin of replication in *E. coli* changes vary little between environments, requiring levels of the key regulator DnaA to be maintained at a constant level. Indeed DnaA is repressed by (p)ppGpp.^57^

Evolutionarily, all these quantitative patterns, from division ‘adders’ to nutrient or ribosome growth laws, could just be spandrels – byproducts of selection on other traits, such as maximal ribosome efficiency. However, cell size matters for many reasons, including the surface-to-volume ratio needed to support nutrient intake, stochastic low-number effects, or transport rates across the cell. If other size-growth patterns would be beneficial, they could also easily be achieved by e.g. adding or removing (p)ppGpp control elements to a few genes. We therefore view the simple passive control above as an elegant and highly efficient way for cells to ensure baseline size adaptation to different conditions.

## Conclusion

Our MARLIN approach combines multi-generational imaging of bacteria in stable, micro-controlled environments followed by precise genotyping of every cell – at a scale of close to a billion cells per day, across a million separate lineages each of which can in principle be a separate strain. In doing so, MARLIN combines the comprehensiveness of genomic screening with the quantitative measurements demanded by biophysics and physiology.

Looking forward, combining MARLIN with deep mutational scanning of RNAs or proteins enables far deeper and more precise phenotypic readouts for library variants than possible with e.g. sort-seq, including single-cell heterogeneity, dynamics and extremely subtle differences in expression. For many biomolecules, such as DNA-binding proteins like activators and repressors, precise quantification and dynamics are essential to understanding the full sequence-function landscape. MARLIN allows us to consider millions of DNA-binding protein variants, systematically connecting binding kinetics to amino acid substitutions.

Expanding out into gene networks, we have seen that MARLIN is uniquely suited to characterizing the quantitative rules emerging from circuits coordinating fundamental cellular processes. Extending this with process-specific reporters for division^63^, replication^64^ and various subcellular structures^65,66^ will uncover additional rules and their molecular implementation. Moreover, MARLIN is also useful for studying synthetic cellular circuits, allowing us to consider not only the individual effects of simple building blocks like promoters and ribosome binding sites, but also far more complex emergent dynamics of networks. Behaviors like bistability, oscillations and excitable dynamics can now be deeply analyzed not just for a handful of systems, but for hundreds of thousands of circuits.

At the broadest cell scale, MARLIN’s combination of immense throughput with phenotypic depth is greatly enabling for physiology, where gene networks interact with each other, biophysical constraints, and the environment to govern global cellular behaviors. By testing hundreds of thousands of variants, we remain agnostic to mechanisms and allow our data to guide holistic, parsimonious physiological models. Moreover, expanding our clustering approach with deeper phenotyping – using more imaging modalities, more reporters and more conditions – we may further functionally annotate the ~1,200 genes^67^ that still have unknown functions in *E. coli*. Finally, using MARLIN to pool commonly-held structured libraries – such as the ASKA, Keio, and various GFP reporter libraries – would enable their routine measurement in combination with different genetic disruptions, reporters and extracellular environments, multiplying our understanding of how major cellular systems interlock. The low cost of running these experiments, combined with the ease of building, sharding and storing pooled libraries, further promises to democratize access.

We believe MARLIN will serve as a tremendous quantitative tool for understanding both the internal mechanics of microbes as well as the synthetic systems we engineer into them. The measurements enabled by MARLIN, coupled with the recent explosion of computational approaches for genomics and model identification, represent a huge step towards the goal of modeling emergent cellular behavior from the cell’s constituent parts.

## Theory Box

### (p)ppGpp Control of Ribosomes

It has previously been shown that ribosome concentration shows positive-linear scaling with growth rate, under varying nutrient conditions and negative-linear scaling under varying concentrations of the elongation-targeting antibiotic chloramphenicol.^52^ In addition, our data suggests that ribosome levels do not vary under growth defects incurred by inhibiting translation initiation. Here, we show how all three of these behaviors may be derived from the (p)ppGpp regulatory model discussed in the main text.

Under this model, (p)ppGpp synthesis is hypothesized to occur in proportion to the concentration of uncharged ribosomes ${\text{R}}_{U}$ competent to bind an uncharged tRNA and stimulate RelA, while (p)ppGpp degradation occurs in proportion to the concentration of charged ribosomes ${\text{R}}_{C}$ with a charged A-site tRNA awaiting translocation. The steady state concentration of (p)ppGpp g is thus the ratio of these concentrations, scaled by a constant k equal to the ratio of rate constants associated with synthesis and degradation:

$$\text{g}=\text{k}\cdot \frac{{\text{R}}_{\text{U}}}{{\text{R}}_{\text{C}}}=\text{k}\cdot \frac{{\text{τ}}_{\text{D}}}{{\text{τ}}_{\text{T}}}$$

During elongation, ribosomes switch from the uncharged to charged state with an average dwell time $\tau_{D}$, followed by switching back into the uncharged state once again after an average translocation time $\tau_{T}$. At equilibrium, elongating ribosomes obey detailed balance and satisfy the condition $R_{U}\tau_{D}^{-1}=R_{C}\tau_{T}^{-1}$, meaning that we may freely substitute ribosome concentrations with the kinetic rates of dwelling and translocation; this form of g will thus allow us to connect (p)ppGpp levels to translation kinetics.

Previously, the behavior of this model was analyzed under variable dwelling time, which was associated with conditions of varying nutrient quality and tRNA charging.^47^ The form of g under variable dwell time was given as a function of the overall translation elongation rate $\varepsilon ={\left(\tau_{D}+\tau_{T}\right)}^{-1}$, with the maximum elongation rate $\varepsilon_{max}=\tau_{T}^{-1}$ associated with the limit ${\text{τ}}_{D}\to 0$:

$${\text{g}}_{\text{Dwell}}\left(\text{R},\text{ε}\right)=\text{k}\cdot \left(\frac{{\text{ε}}_{\max}}{\text{ε}}-1\right)$$

We extend this model to varying initiation and translocation rates by considering model behavior when tRNA charging is already high – i.e. under a reference condition of rapid growth. Under these conditions, dwell time will be primarily limited by the diffusion of ternary complexes^55^ and only weakly dependent on tRNA charge. Therefore, despite the expected increase in tRNA charge under initiation defects, the resulting change in dwell time will be small while the translocation time will be unaffected. As such, (p)ppGpp levels would be expected to remain constant under initiation knockdown:

$${\text{g}}_{\text{Init}}\left(\text{R},\text{ε}\right)={\text{g}}_{\text{ref}}$$

Finally, modeling translocation defects by varying $\tau_{T}$ under a fixed dwelling time ${\text{τ}}_{D}^{0}$, we arrive at the following relationship between (p)ppGpp levels and the elongation rate:

$${\text{g}}_{\text{Elong}}\left(\text{R},\text{ε}\right)=\text{c}\cdot {\text{ετ}}_{\text{D}}^{0}$$

Previously, it was shown that the relationship between ribosome levels R and growth rate λ, under variable dwell time (i.e. obeying $g_{Dwell}$) could be obtained by satisfying a system of equations including the (p)ppGpp repressive function on ribosomes $R\left(g\right)$ and the dependence of growth rate on the ribosome concentration and elongation rates $\lambda \left(R,\varepsilon \right)$. We adopt a similar approach here to model $R\left(\lambda \right)$ under variable initiation and translocation. Motivated by the empirical scaling of (p)ppGpp with ribosome levels^48^ we assume it acts as a simple monovalent repressor of ribosome synthesis:

$$\text{R}\left(\text{g}\right)=\frac{\text{k}}{\text{k}+\text{g}}\cdot \left({\text{R}}_{\max}-{\text{R}}_{0}\right)+{\text{R}}_{0}$$

The growth rate is proportional to the product of the number of active ribosomes $\left(R-R_{inact}\right)$ engaged in translation and the elongation rate:

$$\lambda \left(\text{g}\right)=\text{a}\cdot \text{ε}\left(\text{g}\right)\cdot \left(\text{R}\left(\text{g}\right)-{\text{R}}_{\text{inact}}\right)$$

In the simplifying case where $R_{inact}=R_{0}$ – which appears to be satisfied over most of the range of growth rates examined – solving this system of equations yields $R\left(\lambda \right)$ under both varying initiation and translocation. Combining this with the previously derived form under varying dwelling time results in the following three functions:

$${\text{R}}_{\text{Dwell}}\left(\text{λ}\right)={\text{R}}_{0}+{\text{ε}}_{\max}^{-1}\text{λ}$$

$${\text{R}}_{\text{Init}}\left(\text{λ}\right)={\text{R}}_{\text{ref}}$$

$${\text{R}}_{\text{Elong}}\left(\text{λ}\right)={\text{R}}_{\max}-\frac{{\text{cτ}}_{\text{D}}}{\text{ak}}\text{λ}$$

### Scaling of Unregulated Proteins

We hypothesize that the set of proteins whose activities are limiting for division are only passively regulated by (p)ppGpp, influenced primarily through competition with (p)ppGpp-regulated transcripts for the translation machinery. To formalize how (p)ppGpp regulation will impact this set of proteins and modulate size, we use a conventional proteome allocation framework.^52,68^ In this framework, the cell’s total proteome is partitioned into functionally distinct “sectors,” each occupying a fraction $\phi_{i}$ of the total proteome. Each such sector is further divided into a basal $\phi_{i}^{0}$ and an additional $\Delta \phi_{i}$ allocation capturing any changes from this reference. We will specify the following two coarse-grained proteome sectors: a (p)ppGpp repressed sector $\phi_{C}$ and an unregulated sector $\phi_{NC}$ – which we hypothesize contains the set of limiting division proteins assigned to a sub-sector $\phi_{D}$. Due to the finite size of the proteome, additional $\phi_{C}$ will come at the expense of $\phi_{NC}$ with each sharing a fixed ‘flexible’ portion of the proteome $\Delta \phi^{max}$ according to $\Delta \phi_{C}+\Delta \phi_{NC}=\Delta \phi^{max}$.

To link the fraction of unregulated proteins $\phi_{NC}$ directly to the growth rate, we first introduce the following relationship between the ribosome fraction $\phi_{R}$ – which depends on growth as described previously – and the overall fraction of genes repressed by (p)ppGpp $\phi_{C}$:

$$\Delta \phi_{\text{C}}\left(\text{λ}\right)={\text{f}}_{\text{R}}^{-1}\Delta \phi_{\text{R}}\left(\text{λ}\right)$$

Or equivalently:

$$\phi_{\text{C}}\left(\text{λ}\right)={\text{f}}_{\text{R}}^{-1}\Delta \phi_{\text{R}}\left(\text{λ}\right)+\phi_{\text{C}}^{0}={\text{a}}_{1}\phi_{\text{R}}\left(\text{λ}\right)+{\text{b}}_{1}$$

In other words, we assume that the change in the $\phi_{C}$ sector is linearly proportional to the change in the $\phi_{R}$ sector, according to a constant $f_{R}^{-1}$. We may obtain this condition if we assume that (p)ppGpp regulated genes are controlled according to functions of the same form as the ribosome repression function $R\left(g\right)$ introduced earlier – with the (p)ppGpp binding constant k held constant i.e. assuming (p)ppGpp affinity for RNAP is not strongly impacted by promoter sequence. Combining this result with the constraint of a finite proteome produces the following dependence of $\phi_{NC}$ on growth rate:

$$\Delta \phi_{\text{NC}}\left(\text{λ}\right)=\Delta \phi^{\text{max}}-{\text{f}}_{R}^{-1}\Delta \phi_{\text{R}}\left(\text{λ}\right)$$

We may specify $\Delta \phi_{R}\left(\lambda \right)$ according to the linear relationship of ribosome concentration and growth rate $R\left(\lambda \right)$ under tRNA charge depletion and elongation inhibition, described in the previous section:

$$\phi_{\text{NC}}^{\text{Dwell}}\left(\text{λ}\right)=\phi_{\text{NC}}^{0}+\Delta \phi^{\max}-{\text{f}}_{\text{R}}^{-1}{\text{β}}_{\text{tRNA}}\cdot \text{λ}$$

$$\phi_{\text{NC}}^{\text{Init}}\left(\text{λ}\right)=\phi_{\text{NC}}^{0}+\Delta \phi^{\max}-{\text{f}}_{\text{R}}^{-1}\Delta \phi_{\text{R}}^{\text{Ref}}$$

$$\phi_{\text{NC}}^{\text{Elong}}\left(\text{λ}\right)=\phi_{\text{NC}}^{0}-{\text{f}}_{\text{R}}^{-1}{\text{β}}_{\text{Elong}}\cdot \text{λ}$$

where each β refers to the slope of the corresponding ribosome fraction-growth relationship $\phi_{R}\left(\lambda \right)$. In general, all three equations correspond to a linear form:

$$\phi_{\text{NC}}^{\text{Class}}\left(\text{λ}\right)={\text{a}}_{2}^{\text{Class}}\cdot {\text{λ+b}}_{0}^{\text{Class}}$$

with perturbation-specific slopes and intercepts.

### Negative Hyperbolic Size-Growth Scaling

Finally, to link the abundance of division proteins to cell size, we will need a rough model for cell division. To this end, we will require that the division volume $V_{D}$ scales inversely with the concentration of division proteins $\phi_{D}$ – which being a subset of the unregulated sector $\phi_{NC}$ is equivalent to:

$${\text{V}}_{\text{D}}\propto {1/\phi }_{\text{NC}}=1/\left(1-\phi_{\text{C}}\right)$$

Note that this criterion is generally obtained for the division accumulator family of size control models – while replication-driven models exhibit a more complex dependence of $V_{D}$ on the abundance of the replication-initiating protein and the growth rate. This condition will hold for the average cell volume V, which is proportional to $V_{D}$ for cells growing at steady state – with the exact proportionality set by the conditions of observation e.g. a sample from liquid culture vs trapped lineages (Supplementary Theory).

We integrate our proteome model with this size-concentration relation – substituting $\Delta \phi_{C}$ with $f_{R}^{-1}\Delta \phi_{R}$ – to arrive at the following negative hyperbolic function describing the explicit dependence of cell size on ribosome concentration:

$$\frac{\text{V}}{{\text{V}}_{0}}={\left(1-\text{θ}\cdot \frac{\Delta \phi_{\text{R}}}{\phi_{\text{R}}^{\max}}\right)}^{-1}\;\;\;\;\;\;\;\;\;\;\text{θ}=\frac{{\text{f}}_{\text{R}}^{-1}\phi_{\text{R}}^{\max}}{\phi_{\text{NC}}^{\max}}\;\;\;\;\;\;\;\;\;\;\phi_{\text{i}}^{\max}=\phi_{\text{i}}^{0}+\Delta \phi^{\max}$$

which is equivalent to the relation $V\propto {\left(1-c\phi_{R}\right)}^{-1}$ from the main text. Qualitatively, the parameter θ serves to convert a given percentage change in the ribosome sector, normalized to its maximum size, into an equivalent percentage change in the unregulated sector. This is then converted into a change in volume, with the normalization condition that the minimum volume is achieved when the unregulated sector is maximized $\left(\phi_{NC}^{max}\right)$. Applying the condition-dependent forms of $\Delta \phi_{R}\left(\lambda \right)$ from the previous section to this equation, we arrive at a distinct linear ribosome-growth relationship for each translation perturbation class:

$$\frac{{\text{V}}_{\text{Dwell}}}{{\text{V}}_{0}}\left(\text{λ}\right)={\left(1-\text{θ}\cdot \frac{{\text{β}}_{\text{Dwell}}}{\phi_{\text{R}}^{\max}}\cdot \text{λ}\right)}^{-1}={\left(1-{\text{κ}}_{\text{Dwell}}\cdot \text{λ}\right)}^{-1}$$

$$\frac{{\text{V}}_{\text{Init}}}{{\text{V}}_{0}}\left(\text{λ}\right)={\left(1-\text{θ}\cdot \frac{\Delta \phi_{\text{R}}^{\text{Ref}}}{\phi_{\text{R}}^{\max}}\right)}^{-1}=\frac{{\text{V}}_{\text{Dwell}}}{{\text{V}}_{0}}\left({\text{λ}}^{\text{Ref}}\right)$$

$$\frac{{\text{V}}_{\text{Elong}}}{{\text{V}}_{0}}\left(\text{λ}\right)={\left(\frac{\phi_{\text{NC}}^{0}}{\phi_{\text{NC}}^{\max}}-\text{θ}\cdot \frac{{\text{β}}_{\text{Elong}}}{\phi_{\text{R}}^{\max}}\cdot \text{λ}\right)}^{-1}={\left({\text{α}}_{\text{Elong}}-{\text{κ}}_{\text{Elong}}\cdot \text{λ}\right)}^{-1}$$

Examining the specific case of tRNA synthetase knockdowns – which exhibit positive growth-size scalings of the SMK type – we can see directly how this classic law emerges naturally from our integrated model:

$${\text{V}}_{\text{Dwell}}={\text{V}}_{0}\exp\left({\text{κ}}_{\text{Dwell}}\cdot \text{λ}\right)\exp\left(\frac{1}{2}{\left({\text{κ}}_{\text{Dwell}}\cdot \text{λ}\right)}^{2}+\text{O}\left({\text{λ}}^{3}\right)\right)$$

In other words, our model form can be approximated as exponential in growth rate when the change in $\Delta \phi_{R}$ over conditions is small (e.g. $\kappa_{Dwell}\cdot \lambda$ is small). From this we also see that the deviation from an equivalent exponential fit will be second order in the growth rate, reflecting an increased relative curvature in the negative hyperbolic model.

## Methods

### Barcode Assembly

Our barcode library consists of a set of plasmids, with each plasmid containing a DNA sequence encoding an RNA barcode. Each barcode contains a set of 30 unique binding sites representing a 30 bit word. Each binding site is 20 bp long and consists of one of two possible sequences, “0” or “1”. If the site consists of sequence “1”, it will be complementary to a cognate FISH probe for that site, while if it consists of sequence “0” it will not bind that cognate probe. Site sequences were designed to maximize hybridization rates and minimize crosstalk as described previously for MERFISH secondary probes.^1,21^

Barcodes were generated by cycled ligation assembly (CLA) of pools of 3-bit blocks, each consisting of all 2^3^ possible block sequences.^75^ Both strands of dsDNA coding for each 3-bit block were synthesized as single stranded oligos and pooled to a final concentration of 50 nM for each oligo. These oligos were then phosphorylated by T4 PNK (44 μL oligo pool, 5 μL T4 ligase buffer, 2 μL PNK (NEB, M0201S)). Following this, the phosphorylated oligo blocks were assembled, in proper order, using a mix of “Scaffold Oligonucleotide Connectors” or SOCs, which are unphosphorylated oligos designed with scaffold sequences complementary to the 3 and 5 prime ends of consecutive blocks (4 μL phosphorylation mix, 2 μL 100 nM SOC mix, 2 μL Ampligase buffer, 2 μL 5 μM/μL Ampligase, 10 water). Thermocycling protocol was 95 °C for 2 min, then 60 cycles of 95 °C for 10 s, 60 °C for 30s and 66 °C for 60 s.

To generate barcodes for insertion into plasmids, we diluted the CLA reaction five-fold and amplified full length barcodes using a 100 μL Phusion polymerase (NEB, M0531S0) reaction with 2 μL of diluted CLR template, for 20 cycles, using primers oDE311 and oDE201. An additional 10 μL of 100 μm oDE201 primer (10X) was added to the reaction, which was followed by a single additional thermocycle. Notably, oDE311 contained a random 20bp N-mer sequence such that the final barcode library also included a (20-mer) unique molecular identifier (UMI). 5 μL of this reaction was inspected on an agarose gel for products of the expected length. If non-specific bands were present, reactions would be repeated with fewer cycles. Acceptable products were purified (Monarch PCR & DNA Cleanup Kit, T1030S) and inserted into a oDE202 and oDE253 primer-amplified pDE47 backbone using isothermal assembly (NEB NEBuilder HiFi DNA Assembly Master Mix, E2621L). Assembled barcode plasmids contained a pBR322 origin, a kanamycin resistance gene, and a T7 promoter controlling barcode expression. These assemblies were then purified (Monarch PCR & DNA Cleanup Kit, T1030S), eluted in 6 μL water of which 2 μL was transformed by electroporation into *E. coli* (DH5G Gold Electrocompetent *E.coli*, Genlantis or NEB 10-beta Electrocompetent *E. coli*). 1 mL SOC was added after electroporation and cells were recovered at 37 °C with shaking for 1 hour. The SOC culture was then diluted in 50 mL of LB broth with 50 μg/mL Kanamycin and cultured at 37 °C 220 rpm in a shaking incubator overnight. In parallel, samples of the SOC culture were dilution plated on LB plates with 50 μg/mL Kanamycin to check for adequate transformation efficiency (>10 million CFU/mL). The following day, DNA from the liquid culture was extracted (GeneJET Plasmid Midiprep Kit, K0482) producing the barcode library.

### sgRNA Cloning and Library Assembly

To create a mismatch-CRISPRi library targeting essential genes, we began by selecting all sgRNA sequences in a previous genome-wide CRISPRi screen in *E. coli*.^26^ We considered as essential all genes which exhibited a log fold change < −5.57 at the final timepoint in the original screen or were annotated as essential in a separate TraDIS screen.^25^ For each initial sgRNA, the first member of a mismatch series was generated by mutating the 5’ base pair of the targeting sequence. The second member was then made by further mutating this single-mismatch sequence at the base pair just 3’ to the original mutation. This was continued up to 10 consecutive mutations to generate a total of 11 sgRNAs per target site. 40 control sequences following the design rules of the original CRISPRi screen^26^ and containing less than 8bp of complementarity to any sites in the genome were also included, as well as a handful of sgRNAs from the previous screen targeting non-essential genes.

sgRNA targeting sequences were ordered as an oligo pool from Twist, resuspended to a final (total) concentration of 100 nM in TE buffer. The pool was amplified using a 50 μL Phusion polymerase (NEB, M0531S0) reaction with 1 μL of a 1:250 dilution of the 100 nM oligo pool and oDE418/oDE433 primers, for 8 cycles. The PCR product was purified (Monarch PCR & DNA Cleanup Kit, T1030S) and subsequently digested using BbsI-HF (NEB, R3539S) for 30 mins at 37 °C. 1 μL of 5M NaCl to condition the buffer for a subsequent digestion with BsmBI-v2 (NEB, R0739S) for 30 mins at 55 °C. After heat inactivation at 80 °C for 20 mins, the digested product was purified with a low molecular weight protocol (Monarch PCR & DNA Cleanup Kit, T1030S).

The sgRNA plasmid backbone (pDE93) contains a constitutive sgRNA expression cassette, a pBR322 origin and a Kanamycin resistance gene. This plasmid was digested with BbsI-HF (NEB, R3539S) and CIP treated (NEB, M0525S) to prevent backbone relegation at 37 °C for 1 hour. After heat inactivation at 80 °C for 20 mins, the digested product was run on an agarose gel and the band at the expected backbone size gel purified (Monarch DNA Gel Extraction Kit, T1020L). The digested sgRNA library was then ligated into the plasmid backbone using a 50–100 μM T4 DNA ligase reaction (NEB, M0202S) at RT for 30 mins. The reaction was then purified (Monarch PCR & DNA Cleanup Kit, T1030S) and eluted in 7 μL of water, of which 2 μL was transformed into electrocompetent *E. coli*, recovered in SOC, cultured overnight, and the library was extracted according to the same protocol as with the barcode library.

### Barcoding Library

To assemble our variant libraries with barcodes, we digested both our variant library, barcode plasmid library and a destination vector with BsaI-HFv2 (NEB, R3733S) for 1 hour at 37 °C. The digests were then run on an agarose gel and bands were gel purified corresponding to the cassettes of the two libraries and the backbone of the destination plasmid (p15A ori, Chlor-R). Finally, the two libraries were combinatorially ligated using a 40 μL T4 DNA ligase reaction at RT for 30 mins, purified and eluted in 10 μL. 1 μL of this elution was transformed into electrocompetent *E. coli* with the relevant genetic background and recovered in SOC according to the same protocol as with the barcode library.

To test transformation efficiency, the recovery culture was dilution plated on LB plates supplemented with 25 μg/mL Chloramphenicol, cultured overnight at 37 °C and plating efficiency (CFU/mL recovery mix) measured the next day. Transformation and recovery were then repeated, followed by dilution and plating of the recovery mixture with a target of ~10000 CFU/plate, onto 20–30 plates. An additional dilution series was also performed on this day to measure the efficiency of this transformation. Plates were cultured overnight at 37 °C and the library bottlenecked to the desired complexity by solubilizing colonies on a number of plates corresponding to the desired CFUs, in EZRDM (Teknova, M2105) supplemented with 25 μg/mL Chloramphenicol. The OD600 of the resulting culture was measured and further diluted in media to desired OD600 of 5. Finally, the diluted culture was diluted 1:1 in 50% glycerol, separated into 100 μL aliquots and stored at −80 °C for storage. The residual culture plasmid was extracted (GeneJET Plasmid Midiprep Kit, K0482) for later use in sequencing of the barcode to variant map.

### Library Sequencing

According to our procedure, barcodes are randomly ligated to variants, so we use sequencing to establish the lookup table from each barcode to its corresponding variant. Since the length of the variant-barcode cassette is long (>700bp), we used Nanopore long read sequencing to acquire reads spanning the entire construct and leveraged our barcodes to bin reads by library members.

Library plasmid cassettes were first amplified in a 400 μL Phusion polymerase PCR with primers oDE154 and oDE201 for 12–15 cycles, depending on how many cycles were needed to yield sufficient material. PCR products were then purified by AMPure bead cleanup (Beckman Coulter, A63881). Amplicons were then prepared for sequencing using a standard protocol from Oxford Nanopore Technologies (ONT) which included FFPE DNA Repair (NEB, M6630S), End Repair/dA-Tailing (NEB, E7546S), and ligation of adaptors from the ONT SQK-LSK109 ligation sequencing kit. Prepared amplicons were then run on a ONT MinION with either R9.4.3 or R10.3.1 pores. If necessary to achieve adequate depth, multiple sequencing runs were conducted on a single library and the reads pooled.

Raw reads were processed using high accuracy calling (HAC) with the Guppy basecaller. Following this, reads were aligned to a graph genome reference (gfa) specifying the combinatorial structure of our barcodes using GraphAligner.^76^ The alignment, corresponding to a traversal through this barcode graph, provided the barcode contained in each read, allowing us to subsequently group reads based on barcode. Grouped reads were then aligned to a reference using Minimap2^77^, also specified by the graph alignment, and finally used to generate a barcode-variant consensus using Medaka. This analysis was parallelized using the Snakemake^78^ module in Python (https://github.com/paulssonlab/marlin-nanopore), enabling us to scale this approach to large libraries.

### MARLIN Phenotyping

Prior to experiment, 100 μL library aliquots of *E. coli* were inoculated into 5 mL cultures of EZRDM with 25 μg/mL Chloramphenicol and grown at 37 °C 220 rpm on a shaking incubator to saturation (6–8 hours). Prior to loading cells, the microfluidic was primed by injecting ~200 μL of faCellatite BIOFLOAT^™^ solution (faCellitate, F202005) and allowed it to adsorb at RT for 30 mins. 3 mL of saturated library culture was concentrated 10X by spinning in a microcentrifuge at 4000 rcf for 1 min. Supernatant was then collected to leave a residual ~300 μL of liquid before resuspension of the pellet. The concentrated culture was then injected into the microfluidic to load the library. To ensure maximum loading, the microfluidic was spin loaded using a custom microfuge adaptor, at 650 rcf for 30s, loading the cells into chambers in the downward orientation. The orientation of the device in the adaptor was then reversed and spun at 650 rcf for 30 s, to load chambers in the upward orientation. Once loaded, the device is connected to tubing at the inlet carrying growth medium driven upstream of the device by a peristaltic pump (Longer Precision Pump Co. Ltd, T60-S2&WX10–14), while the outlet is connected to a waste line. The device and feeding lines were then transferred to the microscope for imaging.

Imaging was conducted on a Nikon Ti-2 microscope, with the entire stage enclosed in an incubator (Okolabs) to maintain a constant temperature of 30 °C (lDE20, lDE28 and lDE30) or 37 °C (lDE15). The device was imaged using either a Nikon Plan Apo Lambda 20x Ph2 DM air objective with 0.75 NA (lDE15 and lDE20) or a Nikon Plan Apo Lambda 40x Ph2 DM air objective with 0.95 NA (lDE26, lDE28 and lDE30). All images were acquired using a high pixel density (4.5 μm x 4.5 μm pixel size) sCMOS camera (Photometrics Iris 9) to enable adequate sampling of the 20x objective point spread function. We used an LED lamp (CoolLED pE-100) as a white-light source for phase-contrast imaging and a Spectra III mixed LED/Laser light source (Lumencor) for fluorescence imaging. For most experiments, our light path consisted of a multi-band dichroic (409/493/573/652/759 Brightline, Semrock) and a variable emission filter (519/26, 595/33, or no filter), mounted on an emission filter wheel for fast multi-channel imaging (Finger Lakes Instruments HS-625). For imaging the mKate2Hyb marker in lDE20, we used an alternative configuration with a multi-band dichroic (89403, Chroma) and an 642/80 emission filter.

Before imaging, cells in the microfluidic were allowed to proliferate on-scope for a period of 10–14 hours, under constant flow of media. The phenotyping portion of the experiment then proceeded, with all microscope control and acquisition handled by the Nikon ND Elements software. Depending on the phenotype being measured, different numbers of fields of view and image cadences were used. Imaging of GFPmut2 and mKate2Hyb in lDE15 was conducted every 40 mins for a period of 5 hours and 20 mins, over a grid of 855 fields of view (FOVs). Imaging of mKate2Hyb in lDE20 was conducted every 10 mins for a period of 10 hours, over 629 FOVs. Finally, imaging of mVenus and HU-mCherry in lDE26, lDE28 and lDE30 was conducted every 10 mins for a period of 10 hours, over 325 FOVs. In all cases, the Nikon perfect focus system (PFS) was engaged throughout the experiment to maintain the sample in the focal plane. In all 10 hour CRISPRi experiments, aTc (Sigma, #37919) was added to the flask containing the input growth medium to a final concentration of 0.2 μM at the 2 hour mark, in order to induce dCas9 expression.

### MARLIN Genotyping

Genotyping FISH probes complementary to the MERFISH barcode were ordered from IDT, with the probe DNA conjugated at the 5’ end with a six carbon (C6) linker to a di-thiol group followed by another C6 linker conjugated to a dye. Depending on the experiment, we used probe sets utilizing three of the following five dyes: Alexa Fluor 488, Alexa Fluor 555, Alexa Flour 647, Cy5 and Alexa Fluor 750. Probes were received as lyophilized pellets, resuspended to 100 μM using nuclease-free water, split into a number of strip tubes, and re-lyophilized in 2 nmol aliquots. The dry aliquots were stored at −80C for long term storage. 100 μM stocks were prepared from these aliquots by adding 20 μL of nuclease-free water and kept at −20C for less than six months or until exhaustion. Working stocks of probe combinations to be used in the same imaging cycle were prepared by adding 6.66 μL each of three 100 μM probe stocks to 180 μL of readout probe buffer (TE buffer with 0.1% Triton X-100) to a final concentration of 3.33 μM per probe. Working stocks were stored at −20C for less than two months or until exhaustion. Each round of FISH probing uses 15 μL of this working stalk in a 5 mL hybridization buffer (10% Ethylene Carbonate, 0.125% Triton X-100, 40 U/mL Murine RNAse inhibitor and 20 nM DNA probe in pH 7.0 2X SSC).

Genotyping was conducted immediately after the conclusion of phenotype imaging for each library experiment. Cells in the device were induced for barcode expression for a period of ~75 mins by the addition of Cuminic acid to a final concentration of 100 μM in the input growth media. During this time, we performed setup of our automated liquid handling system (see extended methods), which enabled automated selection of FISH reagents in conjunction with image acquisition. We controlled this system, in conjunction with the microscope, from a Jupyter notebook making use of both a custom Python module for this purpose (https://github.com/paulssonlab/marlin) and the Python Micromanager API.^79^ During setup, all liquid lines in this system were cleaned using DI water and inspected for adequate flow. Once barcode induction was concluded, we loaded the liquid handler with our FISH reagents and replaced the growth medium line with the liquid handler line, just upstream of the device inlet. The liquid handler proceeded to perform fixation and FISH imaging over the next 16–20 hours.

During FISH genotyping, we first inject a 4:1:1:4 solution of methanol, acetone, 20X SSC and nuclease-free water (50% MeAc) into the device, followed immediately by a 4:1 solution of methanol and acetone (MeAc) to fix the cells. We allow fixation to proceed for 45 mins, then inject 50% MeAc, followed by the hybridization solution containing the first probe set. After allowing probes to hybridize for 30 mins, we inject an oxygen-scavenging imaging buffer solution consisting of 3mM PCA, 0.03% Trolox, ~0.18 U/mL rPCO, and Trolox-Quinone (>30 μM, prepared according to a previously published protocol^80^) in pH 7.0 2X SSC. Imaging buffer is flowed for an additional 5 mins to clear any bubbles from the device and then FISH imaging proceeds. During each round of imaging, all FOVs phenotyped previously are imaged in phase-contrast for lineage-detection purposes and three additional fluorescence channels, corresponding to each probe dye. After all channels are imaged, we inject a cleavage buffer (33.3 mL TCEP in pH 7.0 2x SSC) containing a reducing agent to break the di-thiol bond linking dyes to DNA probes, eliminating the fluorescence signal from this round of imaging. To continue genotyping, we repeat this protocol from the hybridization step, for the next set of probes. We repeat 10 such cycles, reading out 3 DNA probes in each, for a total of 30 probe readouts, completing the experiment.

### Mother Machine Isolate Imaging

Mother machine experiments with single isolates were performed using the same imaging setup as described in “MARLIN Imaging”. Starter cultures for strains to be loaded into the device were generally prepared 12–16 hours in advance, in the same manner as described in “Strains and Growth Conditions”. All CRISPRi validation strains were imaged according to the same schedule and under the same conditions as described for lDE20.

### Strains and Growth Conditions

Cells were initially inoculated into 5 mL EZ Rich Defined Medium (EZRDM) with appropriate antibiotics in a test tube from a single colony on an agar plate, struck within 2 weeks of the experiment. Cells were then cultured for 14–16 hrs at 37 °C in a shaking incubator (220 rpm), until saturation. Cells were then diluted 10^−7^ into 5 mL EZRDM with appropriate antibiotics, as a pre-culture, and incubated for an additional 10–12 hrs at 30 °C in a shaking incubator (220 rpm). We then measured OD600 for the pre-cultures and proceeded to dilute each into 25 mL or 5 mL of inductive media (EZRDM + 0.2 μM aTc) with or without 1000 μM IPTG, depending on the experiment. Cultures with OD600 over 0.5 were discarded. Induced cultures were subsequently incubated for 6 hrs at 30 °C in a shaking incubator (220 rpm). Cultures with OD600 between 0.1 and 0.4 at this time were further processed to measure the RNA/Protein ratio (see “RNA/Protein Measurement”) and the (p)ppGpp concentration (see “ppGpp/GTP Sample Harvest Protocol”).

To prepare fixed samples for agar pad imaging the culture protocol was similar, with starter cultures and pre-cultures prepared in the same manner. Pre-cultures were then diluted in 2.5 mL of inductive media (EZRDM + 0.4 μM aTc) and incubated at 30 °C in a shaking incubator (220 rpm) for 6 hours. The OD600 of the cultures were measured and the cultures processed if their OD600 was between 0.1 and 0.4. Subsequently, 360 μL of 32% PFA was added to each tube, which was then placed back into the incubator for a total of 30 minutes. Cells were then collected by centrifugation of the fixed culture at 4000 g for 5 mins, at RT in 10 mL falcon tubes, removing the supernatant and resuspending the pellet in 500 μL of PBS, this centrifugation step was repeated with a resuspension volume of 500 μL and then once more with a final resuspension volume of 250 μL. Samples were then stored at 4 °C until staining and imaging.

## Extended Data

**Extended Data Fig. 2:**
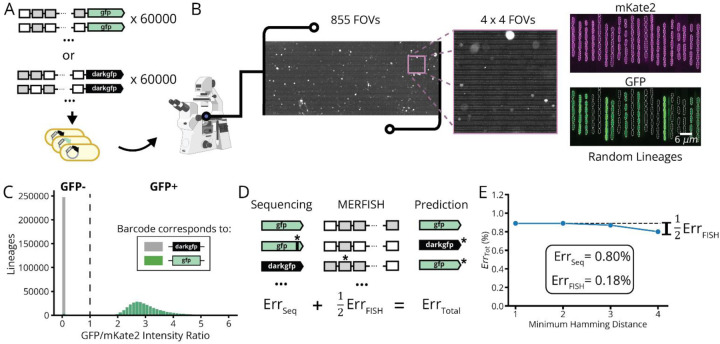
Barcode Error Rate Measurement. **a,** A barcoded library (lDE15) of two fluorescent protein variants, GFP (i.e. GFPmut2) and DarkGFP, was measured by MARLIN to estimate our barcode error rates. **b,** During imaging of lDE15, we measured the fluorescent signal from both a constitutively expressed fluorescent protein encoded on the chromosome (pRpsL-mKate2Hyb) and the constitutively expressed GFP variant expressed on the lDE15 plasmid, every 15 minutes for 6 hours. **c,** Histogram of the average GFP/mKate2 intensity ratio for each lineage in the experiment. We called the lineage as being GFP positive if this ratio was above 1 (dotted line). **d,** Decomposition of total experimental error into contributions from library sequencing and MERFISH readout. Note that the FISH contribution is halved since barcode assignment errors will predict an incorrect GFP variant roughly half the time. **e,** Estimating error contributions by increasing error robustness of barcodes. Computationally filtering lDE15 data for sub-libraries composed of well-separated barcodes with hamming distances of up to four should eliminate the FISH contribution to error. The marginal difference of ~0.09 indicates that the FISH error contribution is ~0.18%. See Supplementary Methods for error estimation details.

**Extended Data Fig. 3:**
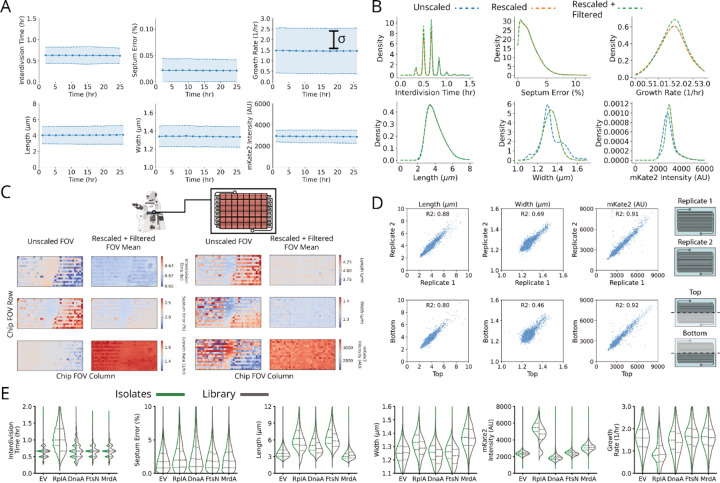
Phenotypic Quantities in MARLIN-CRISPRi Experiments are Highly Reproducible. **a,** Mean and standard deviation estimates for physiological parameters over time for 26 hours of growth of DE32. Values are computed for each 2-hour time window. **b,** Kernel density estimates for distributions of measured physiological parameters without correction (blue line), with trench-wise baseline rescaling (orange line) and with both rescaling and filtering based on growth characteristics (green line). See Supplementary Methods for details. **c,** Mean values of physiological parameters within each FOV across the chip using uncorrected or rescaled and filtered values. **d,** Top, relationship between cell length, width or mKate2 intensity, for each sgRNA in replicate lDE20 experiments. Points correspond to average values in the period from 5 to 8 hours post-induction, for the same sgRNA in each experiment. Bottom, relationship between cell length, width or mKate2 intensity, for each replicate sgRNA in the top or bottom half of the microfluidic. **e,** Distributions of measured physiological parameters in the period from 5 to 8 hours post-induction, for the indicated CRISPRi knockdowns. Distributions correspond to either values determined from the library experiment (right, grey) or from corresponding clonal isolates (left, green). Dotted lines indicate the quartiles of the distributions.

**Extended Data Fig. 4:**
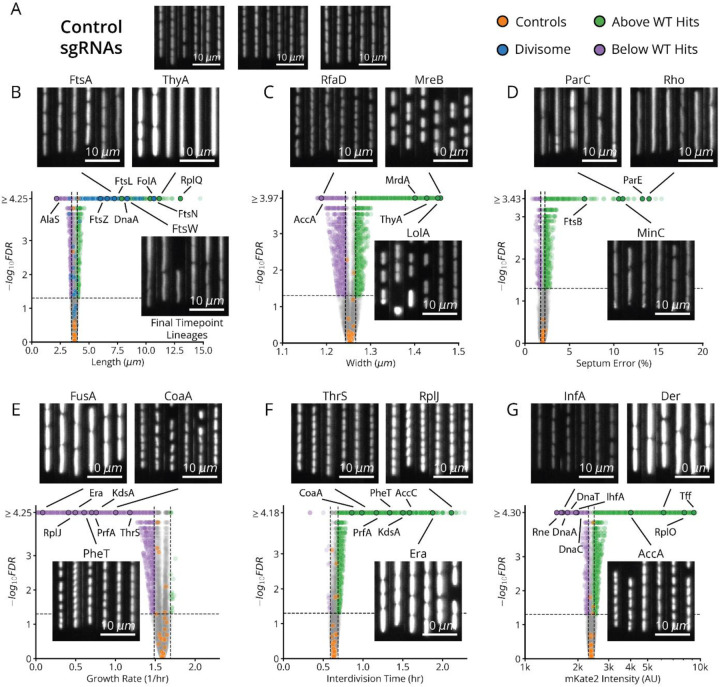
Volcano Plots of 6 Physiological Quantities After CRISPRi Knockdown. **a,** Sample lineages of EV control strains, after 8 hours of CRISPRi induction in our mismatch-CRISPRi library (lDE20). **b,** Volcano plot of cell lengths measured between 5- and 8-hours post-induction, for each sgRNA in lDE20. The horizontal line corresponds to FDR=0.05 and the vertical lines indicate three standard deviations above and below the distribution of control sgRNAs. Sample lineages are shown of representative strains, imaging the cytoplasmic pRpsL-mKate2Hyb marker. **c-g,** Similar volcano plots also included for cell width (**c**), septum placement error (**d**), growth rate (**e**), interdivision time (**f**), and mKate2 intensity (**g**). Consistent with previous reports, depletions targeting members of the cell wall synthesizing Elongasome (*mrdAB*, *mreBCD*, *rodZ*) exhibit an increase in width of >0.1 μm relative to EV controls.^30,71,72^ Also similar to previous reports^31,32^, depletions targeting the division localization factor *minC* and nucleoid partitioning *parCE* system exhibit an increase in septum placement error of >10% relative to EV controls.

**Extended Data Fig. 5:**
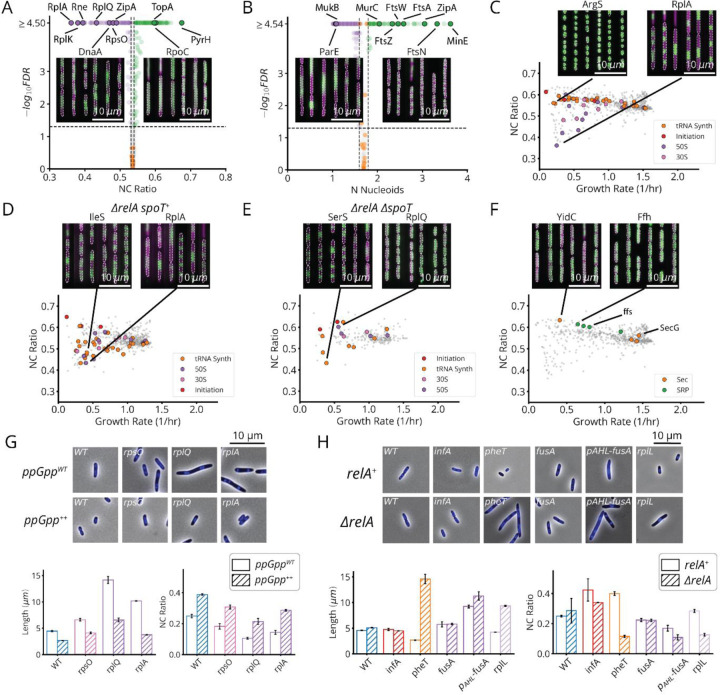
Nucleoid Compaction is Associated with Translation Elongation Defects, without RelA Activation. **a,** Volcano plot of the nucleoid to cell area (NC) ratio, measured between 5 and 8 hours post-induction, for each sgRNA in our HU-mCherry library (lDE26). The horizontal line corresponds to FDR=0.05 and the vertical lines indicate two standard deviations around the distribution of control sgRNAs. Sample lineages are shown of representative strains, where indicated. The cytoplasmic pRpsL-mVenus marker is shown in magenta and the nucleoid-localized HU-mCherry marker is shown in green. **b,** Volcano plot of the number of nucleoids in the cell. **c,** Relationship of NC ratio to growth rate, for all sgRNAs in lDE26. Indicated dots are genes coding for tRNA synthetases (tRNA Synth), translation initiation factors (Initiation), the 50S ribosomal subunit (50S) or the 30S ribosomal subunit (30S). **d,** Same measurement as in panel c, for each sgRNA in our *ΔrelA* library (lDE28). **e,** Same measurement as in panel c, for each sgRNA in our *ΔrelA ΔspoT* library (lDE30). **f,** Same measurement as in panel c. Indicated dots are genes coding for the Sec translocation complex (Sec) or the signal recognition particle (SRP). **g,** Cell length and NC ratio, as measured by DAPI staining, in CRISPRi strains depleted for the indicated gene products, WT denoting the EV sgRNA control. Top, snapshots of the indicated isolates imaged by phase contrast (greyscale) and for fluorescent DAPI stain (blue). *ppGpp*^*WT*^ indicates cells uninduced for the hyperactive (p)ppGpp synthase RelA* and *ppGpp*^*++*^ indicates cell cultured with +25uM IPTG to induce RelA* expression, resulting in ectopic synthesis of (p)ppGpp. Bottom, length and NC ratio measurements of the same strains. Error bars denote 2σ_SEM_ range. **h,** Same measurements as panel g. *ΔrelA* indicates the knockdown was performed in a *ΔrelA* background, while *relA*^*+*^ indicates an intact locus. p_AHL_-fusA denotes the AHL induction strain introduced in Fig. 3e, in which *fusA* was depleted according to the same protocol as in Fig. 3f. Note the difference between this strain and *fusA*, which likely owes to disrupted expression of the downstream *tufA* gene.

**Extended Data Fig. 6:**
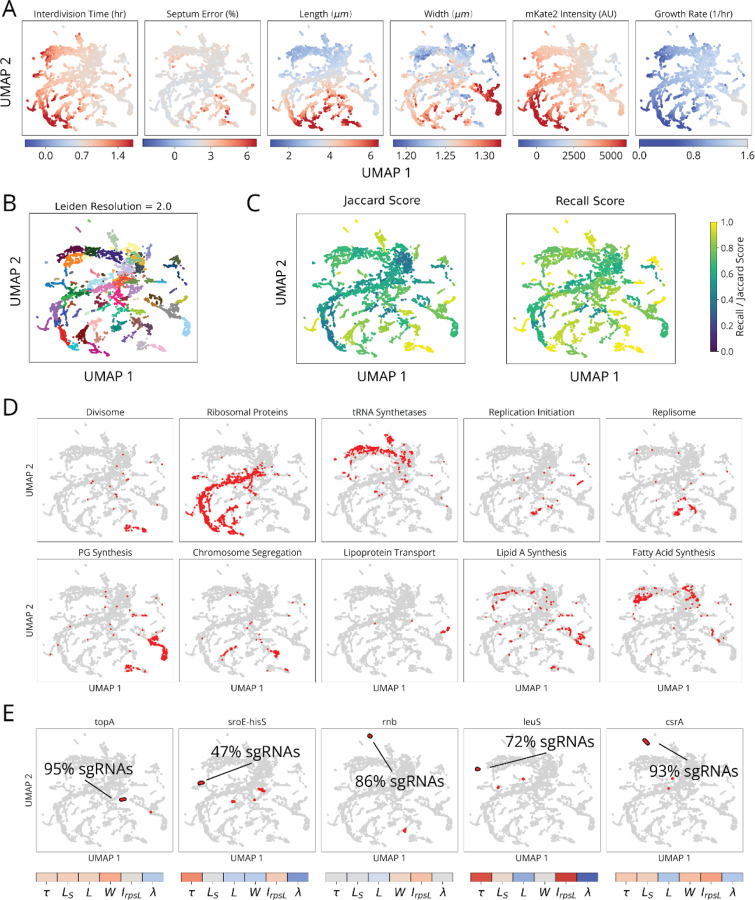
Clustering and Phenotype Distributions in MARLIN-CRISPRi Library. **a,** Distribution of measured parameters in our mismatch-CRISPRi library (lDE20). Colors on UMAPs indicate whether sgRNA variants on the UMAP take an average parameter value above (red), below (blue) or at the value of the sgRNA EV controls (white). **b,** Clusters determined by the Leiden community detection algorithm^73^, indicated by color, with a resolution of 2. **c,** Cluster robustness at different resolutions, determined from 10% jackknife resampling of Leiden clusters and expressed as either the average Jaccard score or the average recall score between a cluster and the most similar cluster in each resampling. **d,** Distribution of different gene groups across lDE20 UMAP, indicated in red. **e,** Top, distribution of different genes or operons across lDE20 UMAP, indicated in red. Points outlined in black correspond to Leiden clusters containing primarily the indicated gene or operon. The percentage corresponds to the fraction of sgRNAs targeting the indicated gene/operon that appear in the selected cluster. Bottom, the average phenotype of the indicated cluster, using the same scaling as in panel a.

**Extended Data Fig. 7:**
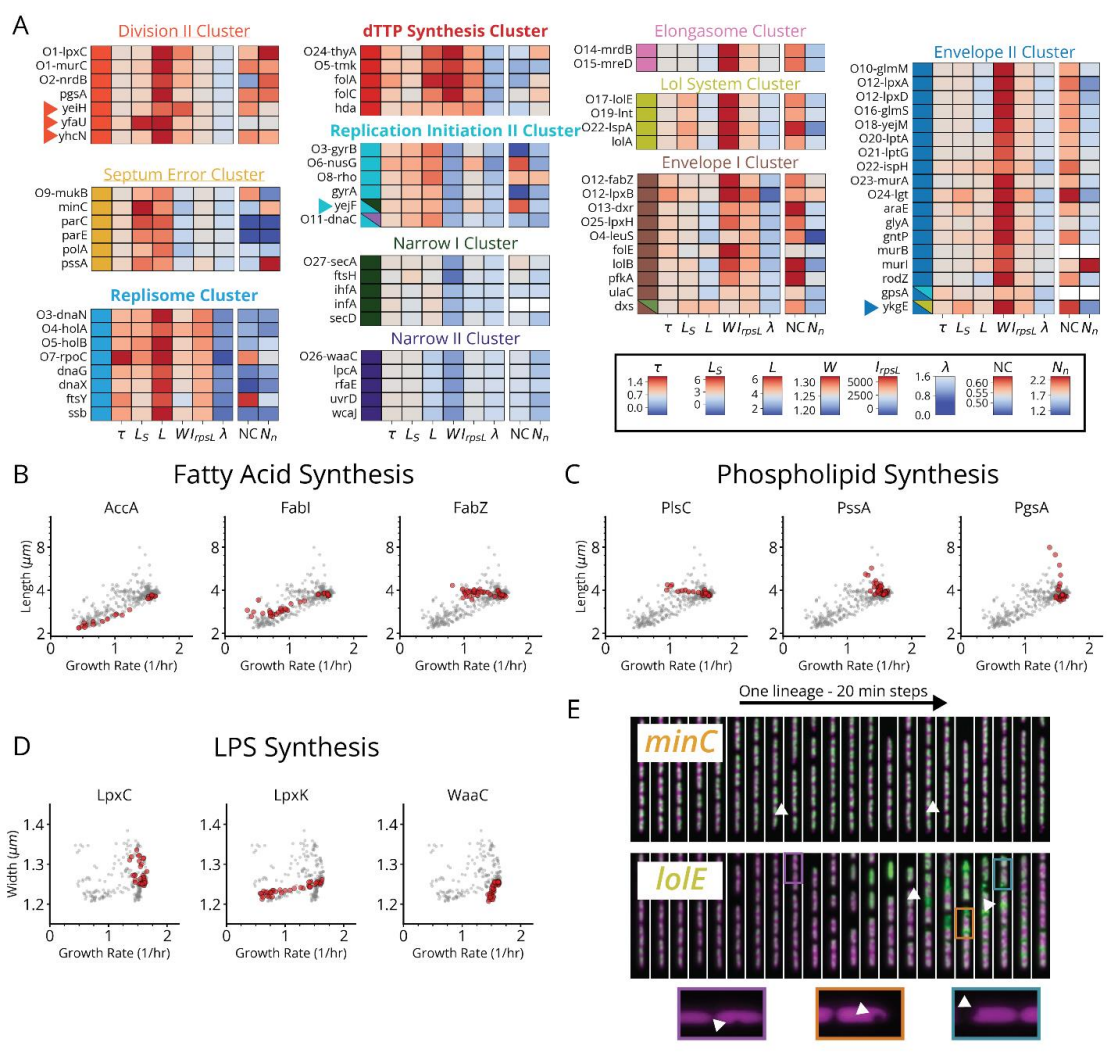
Extended Cluster Heatmaps and Select Scaling Phenotypes for Genes Involved in Fatty Acid, Phospholipid and LPS Synthesis. **a,** Heatmap of cluster phenotypes. Underlined cluster names indicate the division supergroup, bold names indicate the replication supergroup, and plain names indicate the envelope supergroup. Arrows indicate genes mentioned in the text. The leftmost column color denotes cluster membership, right columns display the average value of each quantity at the end of the experiment, for each gene. Same scale as in Fig. 5e. Entries beginning with O denote polycistronic operons, where the subsequent gene name indicates the last gene in a consecutive block all belonging to the indicated cluster. **b-c,** Semi-log plots of average growth rate vs length, measured between 5- and 8-hours post-induction, for sgRNAs in our mismatch-CRISPRi library (lDE20) targeting the indicated genes (red) involved in phospholipid and fatty acid synthesis (grey). **d,** Semi-log plots of average growth rate vs width, measured between 5- and 8-hours post-induction, for sgRNAs in our mismatch-CRISPRi library (lDE20) targeting the indicated genes (red) involved in LPS synthesis (grey). **e.** Representative kymographs of *minC* and *lolE* knockdowns, with the cytoplasm in magenta and nucleoid in green, as in the diagram in Fig. 5f. Top, knockdown, arrows indicating asymmetric divisions. Bottom, kymograph of a *lolE* knockdown, arrows indicating extracellular HU-mCherry resulting from lysis. Insets highlight instances of cytoplasmic invagination and plasmolysis.

**Extended Data Fig. 8:**
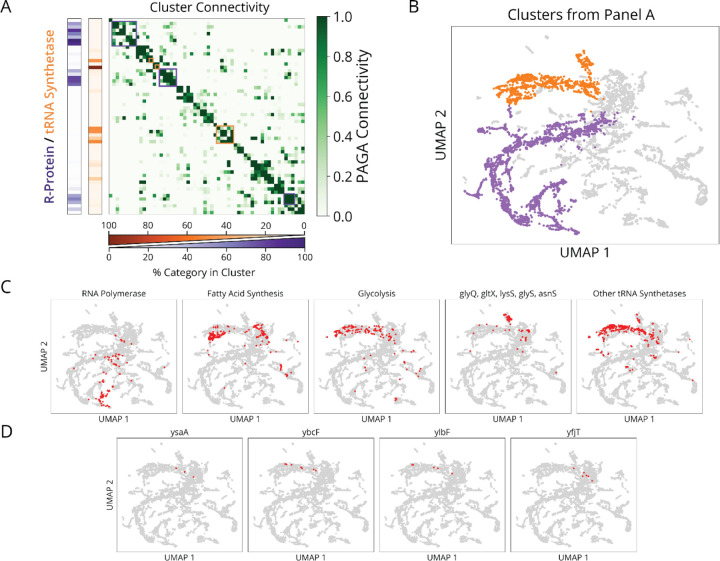
Ribosomal Protein and tRNA Synthetase Clusters are Highly Interconnected. **a,** Left, heatmap indicates the percentage of each cluster composed of either ribosomal proteins (purple) or tRNA synthetases (orange), with each row corresponding to a cluster in our mismatch-CRISPRi library (lDE20). Right, heatmap values indicate statistical connectivity between clusters using the PAGA connectivity metric^74^. Rows are matched to the same clusters as in the left heatmap. Boxed cluster groups indicated highly inter-connected groups enriched for either ribosomal proteins (purple) or tRNA synthetases (orange). **b,** Highlighted UMAP indicating the inter-connected cluster groups corresponding to the boxes in panel a, with the same color scheme. **c,** Distribution of different gene groups across lDE20 UMAP, indicated in red. **d,** Distribution of different genes of unknown function across lDE20 UMAP, indicated in red.

**Extended Data Fig. 9:**
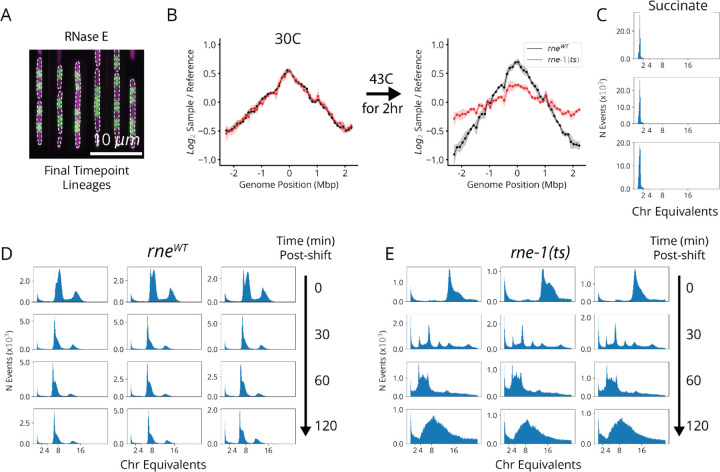
*rne-1(ts)* Cells Rapidly Lose Initiation Activity at a Restrictive Temperature. **a,** Representative lineages depleted for *rne* after 8 hours of CRISPRi induction in our HU-mCherry library (lDE26). The cytoplasmic pRpsL-mVenus marker is shown in magenta and the nucleoid-localized HU-mCherry marker is shown in green. **b,** Marker frequencies based on deep gRNA sequencing for 100kb bins across the *E. coli* genome, with the reference coordinate set to *oriC*. Frequencies normalized to reference measurements from non-replicating cells grown to stationary phase in MBM+0.2% Succinate at 37 °C. Measurements before (left) and after (right) shifting either *rne*^*WT*^ (black) or *rne-1(ts)* (red) cells from 30 °C to 43 °C for 2 hours, with shaded area indicating ±2σSEM based on three biological replicates. **c,** Triplicate flow cytometric measurement of PicoGreen-stained DNA after replication runout for *rne*^*WT*^ cells grown to stationary phase in MBM+0.2% Succinate at 37 °C, used to determine fluorescence intensity of a single chromosome. Chromosome equivalents reported as various multiples (2,4,8,16) of this value. **d,** Same as panel c, for *rne*^*WT*^ cells before (0 mins) and at various times after a shift from 30 °C to 43 °C. **e,** Same as panel d, for *rne-1(ts)* cells.

**Extended Data Fig. 10:**
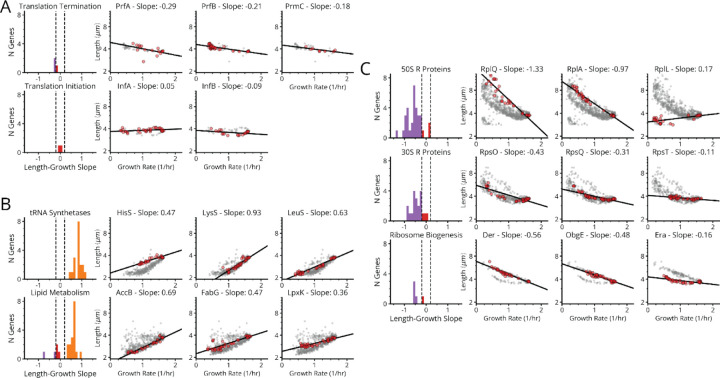
Growth-Size Scaling Analysis for Translation-Related Knockdowns. **a,** Left, histograms of log2 length vs growth rate slope for indicated gene groups including translation termination and initiation factors. Same color scheme for slopes as in Fig. 3b. Right, select log2 length vs growth rate linear fits to groups of sgRNAs targeting single genes belonging to the same gene class. **b,** Same as in panel a, with indicated gene groups including tRNA synthetases and lipid synthesis factors. **c,** Same as in panel a, with indicated gene groups including ribosomal proteins and ribosome biogenesis factors.

**Extended Data Fig. 11:**
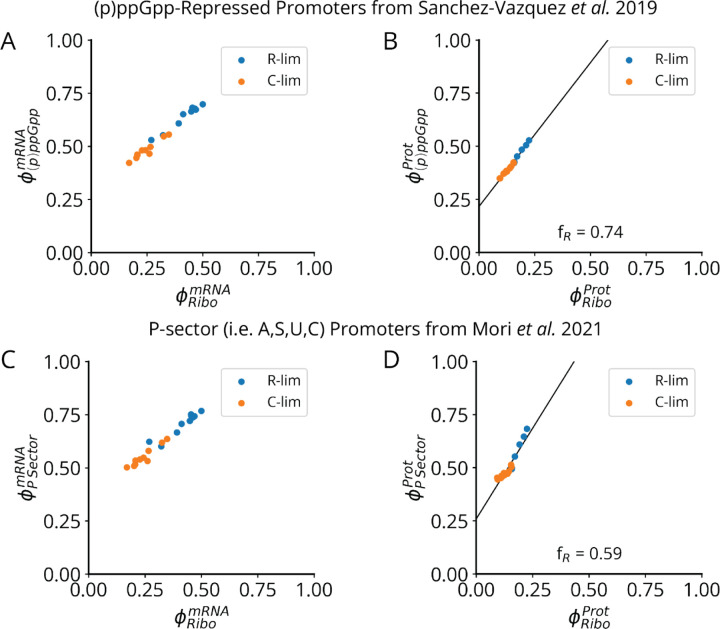
Ribosomal Protein Expression Varies Linearly with the (p)ppGpp Regulon. **a,** Measured ribosomal protein transcriptome fraction vs (p)ppGpp-repressed transcriptome fraction, under limitation of glucose import by PtsG titration (orange points) or translation inhibition by sub-lethal chloramphenicol (blue points). Transcriptome data from Balakrishnan and Mori et al. 2022.^61^ List of (p)ppGpp repressed promoters from Sanchez-Vazquez et al. 2019.^57^ **b,** Measured ribosomal proteome fraction vs (p)ppGpp-repressed proteome fraction, with the same conditions as in panel a. The ribosome fraction of the variable component of the (p)ppGpp regulon f_R_ – defined in the Theory Box – is measured as the slope of this relationship. Proteome data from Mori et al. 2021.^60^ **c-d,** Same plots as panels a-b, but for genes belonging to proteome sectors – identified in Mori et al. 2021^60^ – which are co-regulated with ribosomal proteins (i.e. the A,S,U,C sectors), which are collectively referred to as the (p)ppGpp-repressed or “P-sector”.

**Extended Data Fig. 12:**
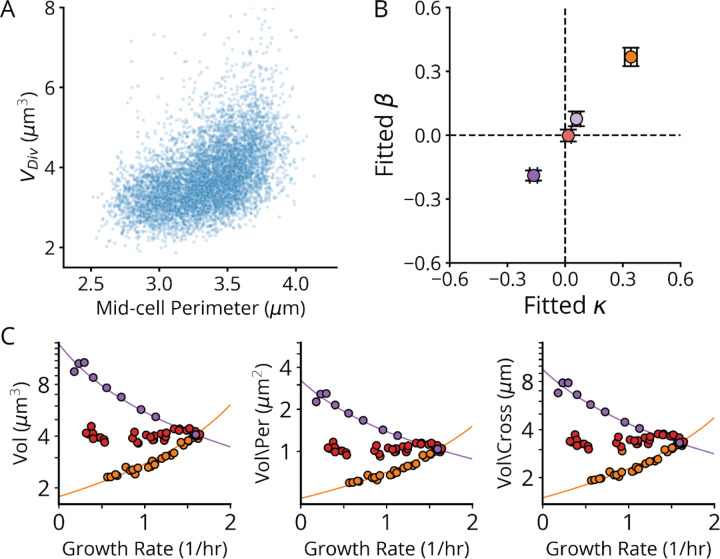
Strict Proportionality of Ribosome and Volume Scaling Constants. **a,** Single-cell measurements of division volume vs mid-cell perimeter at division, between 6–8 hours post-induction in lDE26. Points are only shown for library variants targeting members of the Rod complex (*mrdA*, *mrdB*, *mreB, mreC, mreD, rodZ*) or UDP-GlcNAc synthesis (*glmS*, *glmM*, *glmU*), which primarily impact cell width through reduced cell wall synthesis. **b,** Measured ribosome-growth scaling constants β vs volume-growth scaling constants κ, for *pheT* (orange), *infA* (red), *rplL* (light purple) and *fusA* (dark purple) knockdown series. Derived from scaling data in Fig. 5e,f. See Theory Box for definitions of β and κ. **c,** Same fit of volume vs growth rate as in Fig. 7d (left) as well as equivalent fits where volume is normalized by the measured mid-cell perimeter (center) or the mid-cell cross section (right).

## Supplementary Material

Supplement 1

Supplement 2

## Data Availability

Raw sequencing reads for Nanopore and NGS libraries have been deposited in the NCBI BioProject database under accession number PRJNA1205775. Processed data will be deposited on Zenodo (https://doi.org/10.5281/zenodo.14537796) upon submission. An archived release of the TrenchRipper module version used for this publication is also available on Zenodo at https://doi.org/10.5281/zenodo.14552982. Other source code for image analysis, sequencing analysis, and figure generation are available on GitHub (https://github.com/paulssonlab/2025_Eaton_optical-pooled-screen). Raw or processed image data are available upon request.
